# Characterization of a FOXG1:TLE1 transcriptional network in glioblastoma‐initiating cells

**DOI:** 10.1002/1878-0261.12168

**Published:** 2018-04-27

**Authors:** Rola Dali, Federica Verginelli, Albena Pramatarova, Robert Sladek, Stefano Stifani

**Affiliations:** ^1^ Department of Neurology and Neurosurgery Montreal Neurological Institute McGill University Montreal Canada; ^2^ McGill Center for Bioinformatics McGill University Montreal Canada; ^3^ Departments of Human Genetics and Medicine McGill University Montreal Canada; ^4^ Present address: Laboratory of Cancer Stem Cell Research Candiolo Cancer Institute FPO‐IRCCS Candiolo Italy

**Keywords:** CHAC1, ChIP‐Seq/RNA‐Seq, FOXG1, glioblastoma, Groucho/transducin‐like Enhancer of split, NOTCH

## Abstract

Glioblastoma (GBM) is the most common and deadly malignant brain cancer of glial cell origin, with a median patient survival of less than 20 months. Transcription factors FOXG1 and TLE1 promote GBM propagation by supporting maintenance of brain tumour‐initiating cells (BTICs) with stem‐like properties. Here, we characterize FOXG1 and TLE1 target genes in GBM patient‐derived BTICs using ChIP‐Seq and RNA‐Seq approaches. These studies identify 150 direct FOXG1 targets, several of which are also TLE1 targets, involved in cell proliferation, differentiation, survival, chemotaxis and angiogenesis. Negative regulators of NOTCH signalling, including *CHAC1*, are among the transcriptional repression targets of FOXG1:TLE1 complexes, suggesting a crosstalk between FOXG1:TLE1 and NOTCH‐mediated pathways in GBM. These results provide previously unavailable insight into the transcriptional programs underlying the tumour‐promoting functions of FOXG1:TLE1 in GBM.

**Abbreviations**
BTIC: brain tumour‐initiating cell
ChIP: chromatin immunoprecipitation
DMEM: Dulbecco's modified Eagle's medium
ENCODE: Encyclopedia of DNA Elements
FOXG1: Forkhead Box G1
GBM: glioblastoma
GRG: Groucho‐related gene
IgG: immunoglobulin G
PBS: phosphate‐buffered saline
PWM: positional weight matrix
TLE1: transducin‐like Enhancer of split 1
TMZ: Temozolomide

## 1 Introduction

Glioblastoma (GBM) is the most common and deadly of all brain tumours of glial cell origin (gliomas), with a median survival of less than 20 months (Aldape *et al*., 2015; Louis *et al*., 2007). GBM tumourigenic potential has been partly attributed to the presence of a subpopulation of cells with stem cell‐like properties, termed GBM stem‐like cells or brain tumour‐initiating cells (BTICs) (Dirks, 2006; Lathia *et al*., 2015; Singh *et al*., 2004; Vescovi *et al*., 2006; Yan *et al*., 2013). BTICs fuel tumour growth and initiate tumours after chemotherapy (Chen *et al*., 2012). Elucidating BTIC pathobiology is therefore of the utmost importance for the understanding of gliomagenesis.

We have previously shown that the transcription factor forkhead box protein G1 (FOXG1) contributes to the brain tumour‐initiating ability of GBM patient‐derived BTICs. FOXG1 promotes BTIC self‐renewal potential, inhibits BTIC cell cycle exit and replicative senescence and impedes BTIC progression towards more developmentally mature neural phenotypes (Verginelli *et al*., 2013). FOXG1 also plays similar oncogenic roles in BTICs derived from medulloblastoma, a fast‐growing, high‐grade paediatric brain cancer (Manoranjan *et al*., 2013).

In agreement with these findings, recent work profiling the expression of several forkhead proteins, including FOXG1, as a function of glioma patient survival concluded that forkhead proteins are attractive biomarkers of GBM and warrant further investigation of their roles in gliomagenesis (Robertson *et al*., 2015). Moreover, FOXG1 has been implicated downstream of EGF receptor signalling, one of the most common oncogenic drivers in GBM (Liu *et al*., 2015), further suggesting that FOXG1 is an important effector of GBM tumourigenesis.

In the healthy brain, FOXG1 represses gene expression, at least in part, by forming transcription complexes with Groucho/transducin‐like Enhancer of split (TLE) proteins (Marcal *et al*., 2005; Roth *et al*., 2010; Yao *et al*., 2001). TLE family members are general transcriptional corepressors involved in controlling a variety of cellular processes, including the regulation of cell proliferation and differentiation (Buscarlet and Stifani, 2007; Turki‐Judeh and Courey, 2012; Yuan *et al*., 2017). There are four full‐length TLE family members in mammals, named TLE1‐4, and two shorter isoforms, commonly referred to as Groucho‐related gene product (GRG) 5 and 6. Only full‐length TLE and GRG6 proteins contain a conserved C‐terminal WD40 repeat domain mediating interaction with FOXG1. Full‐length TLE proteins provide a transcriptional corepressor function to FOXG1. In contrast, GRG6 is not endowed with corepressor activity and acts as a dominant‐negative regulator of FOXG1:TLE transcriptional repressor complexes (Marcal *et al*., 2005).

Consistent with the above observations, we have previously shown that FOXG1 physically and functionally interacts with TLE proteins in GBM patient‐derived BTICs, and that TLE knockdown, as well as overexpression of the TLE antagonist GRG6, mimics the effects of FOXG1 attenuation in these cells (Verginelli *et al*., 2013). These findings identify FOXG1:TLE transcriptional complexes as GBM drivers and suggest that the characterization of their transcriptional programs in GBM may contribute to elucidating mechanisms of gliomagenesis and identifying potential targets of therapies for GBM.

FOXG1 and TLE are important for neural stem cell biology and neuronal survival in the healthy brain (Buscarlet and Stifani, 2007; Dastidar *et al*., 2011, 2012; Fasano *et al*., 2009). Thus, they are unlikely drug targets in the fight against GBM. This situation underscores the importance of characterizing the downstream transcriptional targets of FOXG1:TLE complexes as more suitable potential targets for GBM treatment. Here, we sought to identify transcriptional targets of FOXG1 and TLE1 in BTICs using a combination of high‐throughput chromatin immunoprecipitation followed by sequencing (ChIP‐Seq) and RNA sequencing (RNA‐Seq) approaches. The results of these studies provide the first comprehensive genomewide map of FOXG1 and TLE1 targets in GBM cells and identify the gene *cation transport regulator‐like protein 1* (*CHAC1*), a negative regulator of NOTCH signalling and a mediator of apoptosis, as a FOXG1:TLE1 target in GBM.

## 2 Materials and methods

### 2.1 Brain tumour‐initiating cell culture

Two previously characterized GBM patient‐derived BTIC lines, BT048 and BT025 (Cusulin *et al*., 2015; Kelly *et al*., 2009; Verginelli *et al*., 2013), were used. These cells were obtained from Samuel Weiss at the Hotchkiss Brain Institute at the University of Calgary, in Calgary, Alberta, Canada. BTICs were maintained under previously described culture conditions (Verginelli *et al*., 2013).

### 2.2 Lentiviral transduction of brain tumour‐initiating cells

For knockdown studies, bicistronic lentiviral particles expressing either a control, nonsilencing (‘scrambled’) shRNA reagent (catalog No. RHS‐4348) or previously validated (Verginelli *et al*., 2013) shRNA sequences targeting human *FOXG1* (sense sequence #1: 5′‐ATGGGACCAGACTGTAAGTGAA; Clone ID V3LHS_40 7592; sense sequence #2: 5′‐CCAGCTCCGTGTTGACTCAGAA; Clone ID V3LHS_353952) or *TLE1* (sense sequence #1: 5′‐AGCAGTCTCCACTTGGCAATAA; Clone ID V2LHS_18400) were obtained from Open Biosystems (Lafayette, CO). Additional *FOXG1* shRNA sequences were as follows: sense sequence #3: 5′‐CCGTGTTTGTCACTTACAA; Clone ID V3LHS_407593; and sense sequence #4: GAGAATACATTGTAGAATA; Clone ID V2LHS_43017. Low passage number BTICs were transduced at a multiplicity of infection of 5 and were analysed 5 days post‐transduction. Knockdown efficiency was evaluated by western blotting analysis of FOXG1 or TLE1 protein expression as described (Verginelli *et al*., 2013).

### 2.3 ChIP‐Seq

Cultured BTIC spheres were dissociated in Accumax (Innovative Cell Technologies, San Diego, CA, USA; catalog No. AM1) and washed with PBS. Aliquots of 3.3 × 10^7^ cells were suspended in 1% formaldehyde in 10 mL of Dulbecco's modified Eagle's medium for 10 min, followed by incubation in 1 mL of 125 mm glycine for 5 min. Each aliquot was washed twice with PBS and stored at −80 °C until use. ChIP was carried out using the Magna ChIP^T/M^ A/G kit (Millipore Canada, Etobicoke, ON, Canada; catalog No. 17‐10085) according to the manufacturers’ instructions. Each aliquot was sonicated to about 100–500 bp in size using a Diagenode Bioruptor UCD‐300 water bath sonicator using the following settings: 10 s ON followed by 20 s OFF for six sets of 15 cycles each on HIGH. Sonicated DNA was reverse cross‐linked and RNAse treated, then fractionated on agarose gel to confirm DNA size distribution. Antibodies used were rabbit anti‐FOXG1 (Abcam Inc., Toronto, ON, Canada; catalog No. ab18259) at 3 μL per cell aliquot, and rabbit anti‐TLE1 antibody (Nuthall *et al*., 2004; Verginelli *et al*., 2013; Yao *et al*., 2001) at 3 μL per cell aliquot. Rabbit immunoglobulin G (IgG) (Cell Signalling Technology, Danvers, MA, USA; catalog No. 2729) was used as negative control. ChIP products were collected in water and quantified. PCR was used to evaluate ChIP outcome before library preparation using *CDKN1A*/*p21* ^*Cip1*^ promoter primers as positive control (Verginelli *et al*., 2013). Libraries were created from successful large‐scaled ChIP experiments using Illumina's TruSeq Library Prep Kit following instructions in the user manual (Illumina, San Diego, CA, USA). Library size selection was carried out using the PippinPrep kit (Sage Science, Beverly, MA, USA) to select DNA fragments between 200 and 400 bp. Successful libraries were submitted for sequencing on the Illumina HiSeq2000 at 50‐bp single‐read sequencing. ChIP‐Seq experiments were run in two replicates.

### 2.4 ChIP‐Seq bioinformatics analysis

ChIP‐Seq reads were quality controlled and trimmed for adapter sequences using Trim Galore (Babraham Bioinformatics, Cambridge, UK). Filtered reads were aligned to hg38 using Bowtie2 (Langmead and Salzberg, 2012). Peaks were called using MACS14 (Zhang *et al*., 2008) using both IgG or input DNA as control. Putative positional weight matrices were identified using the ‘findMotifsGenome’ module form HOMER (Heinz *et al*., 2010). Peaks were annotated using the ‘BSgenome.Hsapiens.UCSC.hg38’ library from R/Bioconductor. The genomewide peak distribution was assessed using the ‘ChIPpeakAnno’ library (Zhu *et al*., 2010) from R/Bioconductor. ENCODE human transcription factor binding sites (Dunham *et al*., 2012) were downloaded from the UCSC Genome Browser (http://hgdownload.cse.ucsc.edu/goldenPath/hg19/encodeDCC/wgEncodeRegTfbsClustered/wgEncodeRegTfbsClusteredV3.bed) and intersected with FOXG1 or TLE1 peaks using ‘GenomicRanges’ package (Lawrence *et al*., 2013) in R/Bioconductor at a maximum distance of 500 bps.

### 2.5 RNA‐Seq

FOXG1 or TLE1 were silenced in BTICs (1.5 × 10^6^ cells) using previously validated (Verginelli *et al*., 2013) shRNA reagents (sense sequence #1 for either FOXG1 or TLE1; both sequences as defined in section 2.5 above) delivered by lentiviral transduction. Cells were cultured for 5 days after transduction then harvested for protein and RNA isolation. Total RNA was extracted using TRIzol Reagent (Invitrogen, Carlsbad, CA, USA; catalog No. 15596‐026) and sent to the McGill University and Genome Quebec Innovation Centre for quality control, polyA+ selection, library preparation and sequencing on Illumina HiSeq2000 at 100 bp pair‐ended. Two replicates of the RNA‐Seq experiments were performed.

### 2.6 RNA‐Seq bioinformatics analysis

RNA‐Seq reads were quality controlled and trimmed for adapter sequences using Trim Galore (Babraham Bioinformatics). Filtered reads were aligned to hg38 using TopHat (Trapnell *et al*., 2012). Read counts for each gene were carried out using HT‐Seq (Anders *et al*., 2015) using the hg38 refSeq refFlat GTF file accessed on July 2015. Batch effects in the two replicates of the RNA‐Seq experiments were corrected using ComBat from Bioconductor. Differentially expressed genes were analysed using the DESeq package (Anders and Huber, 2010) at an adjusted *P*‐value cut‐off of 0.1. Gene ontology analysis was carried out based on the PANTHER classification system (Mi *et al*., 2013, 2016).

### 2.7 Polymerase chain reaction

PCR primers were ordered from Integrated DNA Technologies (Coralville, IA, USA) and suspended in water to a final concentration of 10 μm. Primers were designed to have a melting temperature near 54–56 °C. ChIP DNA products (1 μL) were diluted to a final volume of 30 μL for each PCR mixture. PCR program was 2 min at 94 °C, then 30 cycles of 94 °C for 30 seconds, primer pair melting temperature for 30 s then 72 °C for 30 s, followed by a final extension phase at 72 °C for 5 min. PCR products were run on 1% agarose gel containing ethidium bromide. The sequences of the oligonucleotides used in PCR experiments are listed in Table S1.

### 2.8 RT‐qPCR

Total RNA was isolated from BTICs using TRIzol reagent and reverse transcribed using Bio‐Rad iScript™ Reverse Transcription Supermix for RT‐qPCR (Bio‐Rad, Mississauga, ON, Canada; catalog No. 170‐8840). qPCR was performed using the Bio‐Rad CFX96 Touch™ Real‐Time PCR Detection System using SsoFast™ EvaGreen Supermix (catalog No. 172‐5201). Expression values were expressed as fold change of *FOXG1* or *TLE1* in silencing shRNA‐transduced cells over nonsilencing shRNA‐transduced cells using β*‐ACTIN* as a control by the Comparative CT Method of analysis (means of three technical replicates). The sequences of the oligonucleotides used in qPCR experiments are listed in Table S1.

## 3 Results

### 3.1 FOXG1 and TLE1 genomic binding sites in brain tumour‐initiating cells

**Figure 1:**
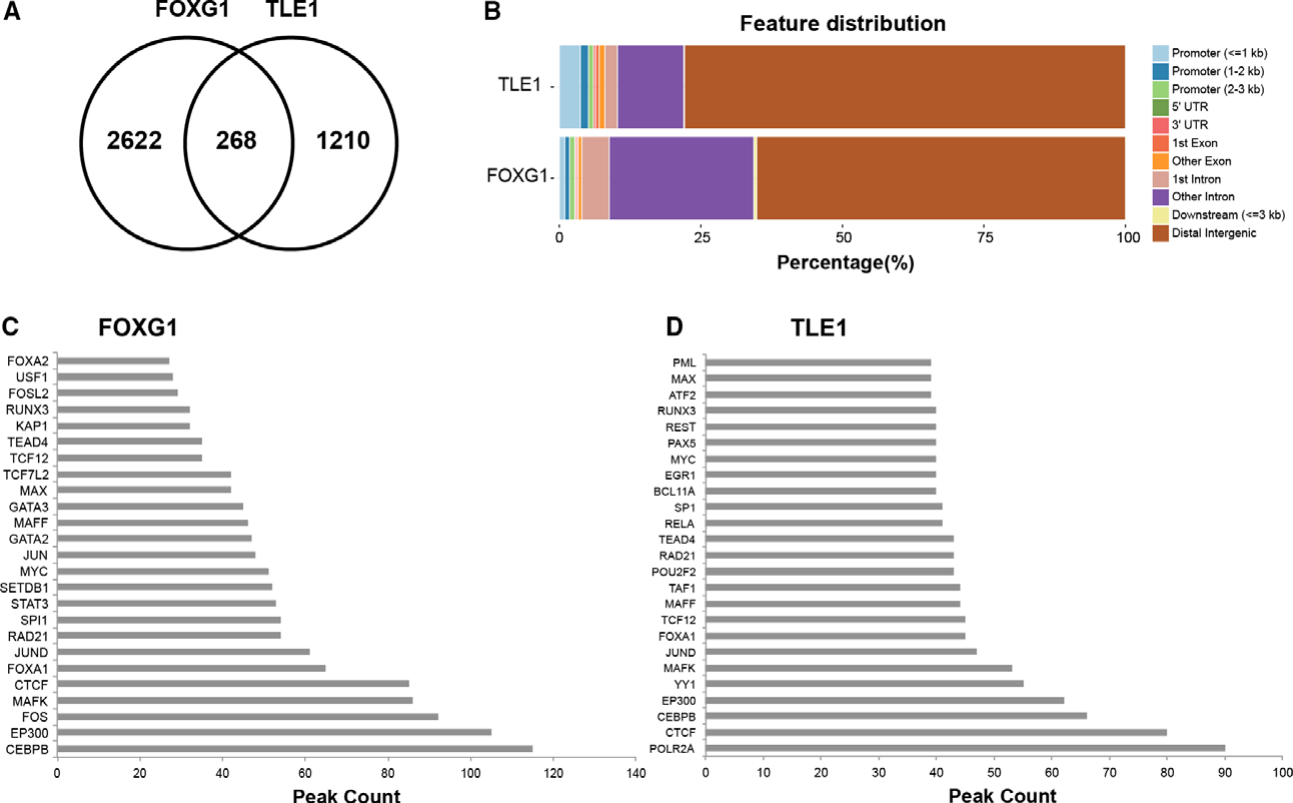
Analysis of FOXG1 and TLE1 ChIP‐Seq experiments. (A) Common FOXG1 and TLE1 ChIP‐Seq peaks. FOXG1 and TLE1 share 268 common regions with a minimum overlap of at least 1 bp. (B) Genomewide distribution of peaks from FOXG1 ChIP‐Seq (bottom bar plot) and TLE1 ChIP‐Seq (top bar plot). (C) Peak count of ENCODE transcription factor ChIP‐Seq peaks overlapping with FOXG1 peaks within a maximum gap of 500 bp. (D) Peak count of ENCODE transcription factor ChIP‐Seq peaks overlapping with TLE1 peaks within a maximum gap of 500 bp.

To identify FOXG1 and TLE1 gene targets in BTICs, large‐scale ChIP experiments for FOXG1 and TLE1 proteins were conducted using the previously characterized BTIC line BT048 (Cusulin *et al*., 2015; Kelly *et al*., 2009). Rabbit IgG was used as a negative control for nonspecific pull‐down products, and input DNA not subjected to ChIP was assessed to control for unequal coverage across the genome. A total of 2890 FOXG1 peaks and 1478 TLE1 peaks were identified using input or IgG as control: of these, 268 peaks were shared (Fig. 1A). To validate the specificity of the FOXG1 ChIP‐Seq experiments, abundant positional weight matrices (PWM) were assessed using HOMER (Heinz *et al*., 2010). Although the PWM of FOXG1 itself is not included in most databases, several binding motifs containing the Forkhead core binding motif were identifiable, including the PWM of FOXA1, FOXP1 and FOXH1. The position of FOXG1 and TLE1 peaks relative to gene annotations was evaluated (Fig. 1B). Over 60% of the FOXG1 peaks and 75% of the TLE1 peaks were located in distal intergenic regions, suggesting long‐range transcriptional function or genomic functions that extend beyond transcriptional regulation. About 20% of peaks for both proteins were close to genes.

To identify putative transcriptional partners, FOXG1 (Fig. 1C) or TLE1 (Fig. 1D) peak coordinates were intersected with genomewide ENCODE‐identified transcription factor peaks: the number of common peaks between the top 25 transcription factors, whose co‐occurrence with FOXG1 or TLE1 is higher than what would be expected by chance, was reported allowing a maximum gap of 500 bp. The top transcription factors overlapping with FOXG1 peaks were CEBPB, CTCF and FOXA1, among others. Amid the top transcription factors overlapping with TLE1 peaks were transcription factors that overlapped FOXG1 peaks, including CEBPB, CTCF, JUND and FOXA1. Known TLE transcriptional partners like TCF12 and RUNX (Buscarlet and Stifani, 2007) were also identified. These findings suggest potential transcriptional interactions involving FOXG1 and TLE1 with other factors previously implicated in GBM, including CEBPB, CTCF and JUND (Ayala‐Ortega *et al*., 2016; Rong *et al*., 2009; Talasila *et al*., 2017).

### 3.2 FOXG1 binds FOXA1 sites

FOXG1 and FOXA1 are members of the same family of transcription factors and share a common DNA‐binding domain known as the forkhead domain. Multiple sequence alignment of both proteins shows a large degree of conservation, especially within the DNA‐binding forkhead domain (Fig. S1A). The PWM bound by FOXG1 shows similarity to the FOXA1 PWM (Fig. S1B), suggesting that FOXG1 and FOXA1 may bind similar genomic regions; in agreement with this, our ChIP‐Seq analysis showed that FOXG1 binds several ENCODE‐identified FOXA1 sites (Fig. 1C). To test this hypothesis further, FOXA1 ENCODE‐identified peaks were tested for FOXG1 binding. ChIP was conducted in triplicates in two characterized BTIC lines, BT048 and BT025. Most tested FOXA1 sites bound FOXG1 in BTICs: two of these sites, *ANAPC10* and *CDKN1B/p27* ^*Kip1*^, are shown in Fig. S1C.

### 3.3 Identification of FOXG1‐ and TLE‐regulated genes with RNA‐Seq

**Figure 2:**
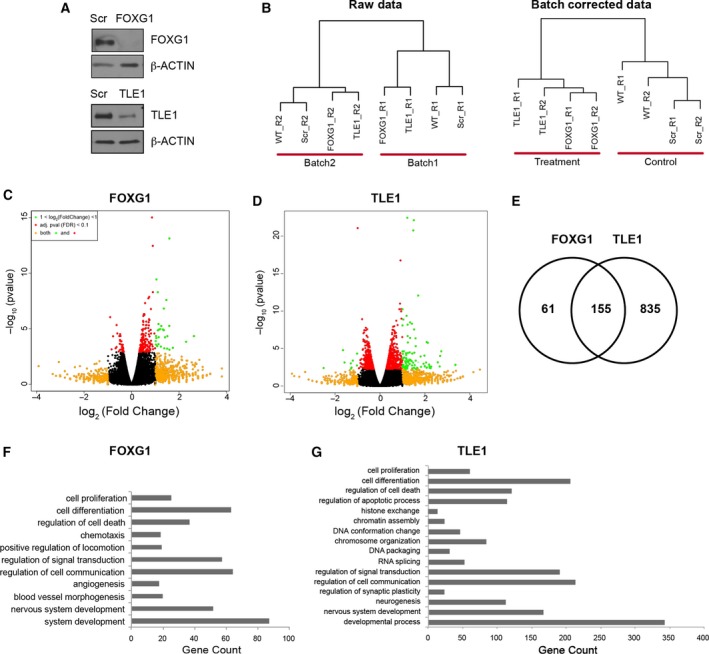
Analysis of RNA‐Seq data following FOXG1 and TLE1 knockdown. (A) Western blotting analysis of FOXG1 and TLE1 protein levels following lentivirus‐mediated delivery of specific shRNA reagents in BTIC line BT048. Scr = scrambled, nonsilencing shRNA control; FOXG1 = shRNA targeting FOXG1; TLE1 = shRNA targeting TLE1. Expression of β‐ACTIN is shown as loading control. (B) Hierarchical clustering of samples before and after batch correction. WT = wild‐type, nontransduced, BT048 cells; Scr = BT048 cells expressing scrambled control shRNA; FOXG1 = BT048 cells expressing shRNA targeting FOXG1; TLE1 = BT048 cells expressing shRNA targeting TLE1. R1 = replicate #1; R2 =  replicate #2. (C) Volcano plot of differentially expressed genes after FOXG1 knockdown. Genes with an adjusted *P*‐value [false discovery rate (FDR)] greater than 0.1 are coloured in orange. Genes with log_2_ (fold change) greater than 1 are coloured in red. Genes with adjusted *P*‐value greater than 0.1 and log_2_ (fold change) greater than 1 are coloured in green. (D) Volcano plot of differentially expressed genes after TLE1 knockdown. (E) Genes differentially expressed following FOXG1 or TLE1 knockdown at an adjusted *P‐*value of 0.1. (F) Selected significant gene ontology categories of differentially regulated genes following FOXG1 knockdown. (G) Selected significant gene ontology categories of differentially regulated genes following TLE1 knockdown.

Next we sought to identify genes whose expression is regulated by FOXG1 and TLE1. BT048 cells were transduced with lentivirus encoding previously validated shRNA sequences targeting *FOXG1* or *TLE1*, or nonsilencing shRNA control (Verginelli *et al*., 2013). After confirming FOXG1 or TLE1 protein attenuation (Fig. 2A), RNA was collected and sequenced. The RNA‐Seq analysis exhibited a batch effect (Fig. 2B left panel), which was corrected using Combat in Bioconductor (Leek *et al*., 2016) (Fig. 2B right panel). Expression analysis identified 216 genes that were differentially regulated following FOXG1 knockdown (Fig. 2C) and 990 genes following TLE1 knockdown (Fig. 2D), with 155 genes in common (Fig. 2E; Table S2). More genes were upregulated than downregulated following FOXG1 or TLE1 knockdown (Fig. 2C, D), consistent with the previously characterized transcriptional repressor function of these proteins. Gene ontology of modulated genes following FOXG1 knockdown, analysed using the PANTHER classification system (Mi *et al*., 2013, 2016), showed several predicted categories, like ‘nervous system development’, ‘cell proliferation’ and ‘cell differentiation’ (Fig. 2F; Table S3). Other categories, such as ‘chemotaxis’ and ‘angiogenesis’, were unanticipated. Gene ontology categories following TLE1 knockdown (Fig. 2G) showed common categories with FOXG1, but also included other groups suggesting a role for TLE1 in chromatin structure, which has been previously proposed (Sekiya and Zaret, 2007).

### 3.4 Integrative analysis of ChIP‐Seq and RNA‐Seq

**Figure 3:**
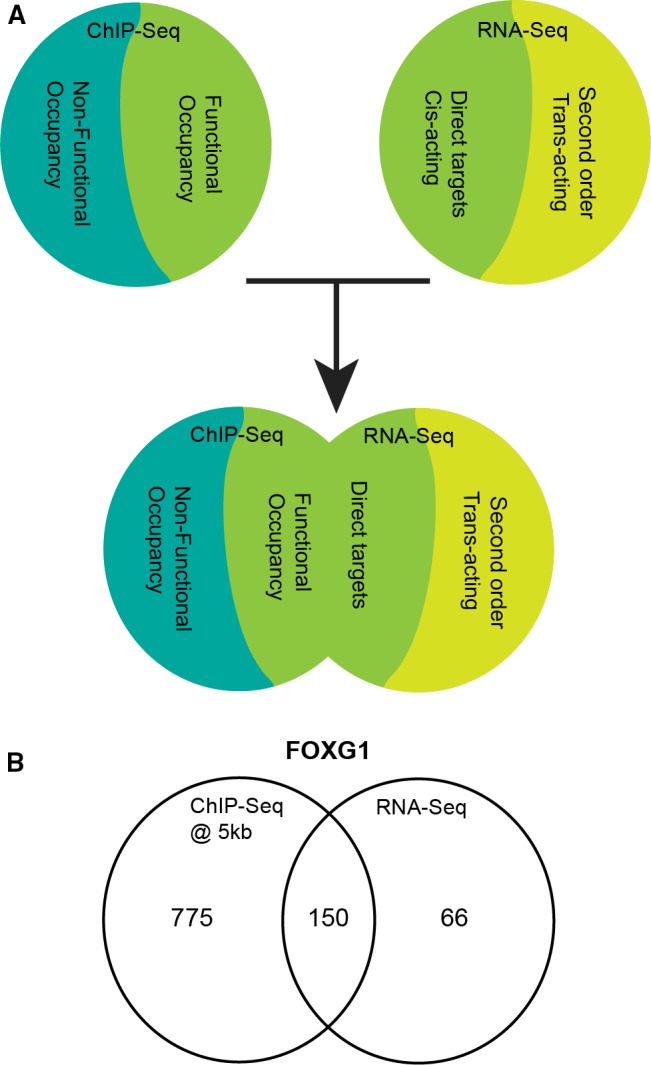
Integration of ChIP‐Seq and RNA‐Seq data. (A) Schematic representation of the integrative analysis of ChIP‐Seq and RNA‐Seq experiments. (B) Gene counts resulting from the integrative analysis of FOXG1 ChIP‐Seq and RNA‐Seq approaches.

To identify FOXG1 direct target genes, we integrated the results of ChIP‐Seq and RNA‐Seq analyses. ChIP‐Seq results contain both functional transcription factor occupancy that leads to transcriptional modulation and nonfunctional occupancy where the transcription factor binds DNA but does not affect nearby genes. RNA‐Seq results contain direct gene targets regulated by the transcription factor of interest but also include second‐order targets that might be modulated by the transcription factor's first‐order targets. Combining ChIP‐Seq and RNA‐Seq results provides a better understanding of the direct targets of a given transcription factor (Fig. 3A). This analysis identified 925 genes that are within 5 kb from a FOXG1 or FOXA1 ENCODE peak, of which 150 had differential gene expression upon FOXG1 knockdown, consistent with the notion that they correspond to direct FOXG1 targets (Fig. 3B, Table S4). In agreement with the role of FOXG1 as a transcriptional repressor, 89% of the 150 identified genes showed increased expression following FOXG1 knockdown. Of note, two of the genes upregulated following knockdown of FOXG1 in BTICs, *DMRTA1* and *EGR2* (Table S4)*,* had been identified previously as high‐probability transcriptional repression targets of mouse Foxg1 in the developing brain (Kumamoto *et al*., 2013). Importantly, 106 of these direct FOXG1 targets were also identified as genes whose expression is modulated following TLE1 knockdown (Table S5; see also Table S2).

**Figure 4:**
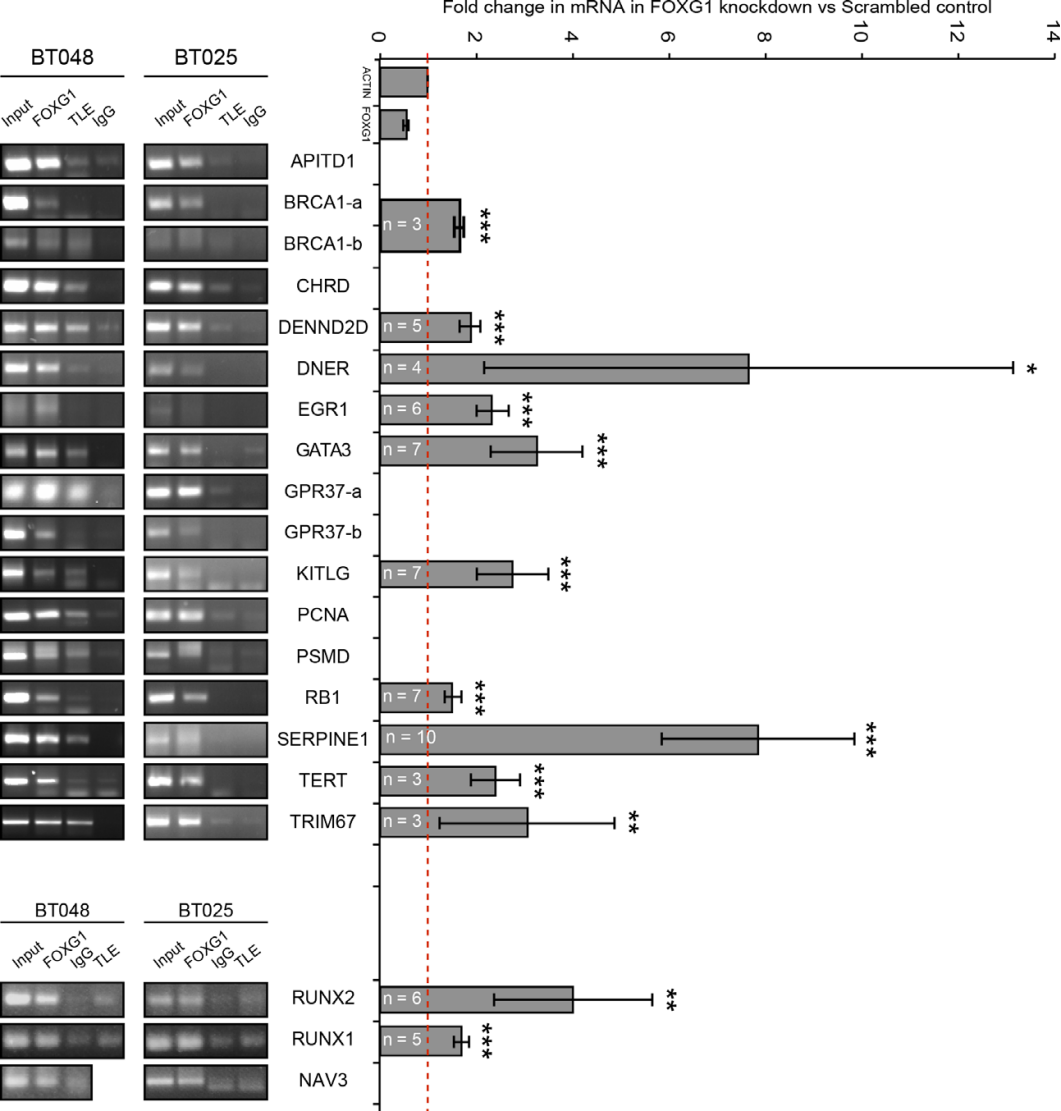
Validation of selected FOXG1 targets by direct ChIP and RT‐qPCR. ChIP analysis of selected FOXG1 or TLE1 binding sites in BT048 and BT025 cells using anti‐FOXG1, anti‐TLE1 or control (IgG) antibodies, as indicated, followed by PCR with primers specific for each region (left panel). Right panel depicts fold changes in gene expression following lentiviral‐mediated FOXG1 knockdown compared to nonsilencing shRNA control in BT048 cells assayed using RT‐qPCR (*P < 0.05; **P < 0.01; ***P < 0.001).

To extend the analysis of FOXG1‐regulated genes, we selected two different categories of candidate FOXG1 transcriptional targets for direct ChIP and RT‐qPCR analysis. These candidates included examples of both genes identified by combined RNA‐Seq and ChIP‐Seq data and genes with upregulated expression following FOXG1 knockdown but located at > 5 kb from nearby ChIP peaks (Fig. 4). Most of these selected candidates had been previously implicated in mechanisms relevant to gliomagenesis, such as regulation of cell proliferation and survival, inhibition of neural cell differentiation and participation in signalling pathways implicated in cancer development. Direct ChIP assays demonstrated that many of the selected sites were occupied by both FOXG1 and TLE1 in both BTIC lines BT048 and BT025 (Fig. 4, left panel). Moreover, most of these target genes displayed increased expression upon FOXG1 knockdown using RT‐qPCR (Fig. 4, right panel). Together, these results identify a number of transcriptional targets of FOXG1:TLE1 complexes in BTICs, providing previously unavailable information on the transcriptional programs regulated by these proteins during gliomagenesis.

### 3.5 *CHAC1* is a FOXG1 and TLE1 target in brain tumour initiating cells

The gene *CHAC1* exhibited the most robust upregulation following both FOXG1 and TLE1 knockdown (Table S2). Moreover, a FOXG1 ChIP peak was identified approximately 8 kb from the *CHAC1* gene (not shown), suggesting that *CHAC1* is a FOXG1 target. Previous studies showed that CHAC1 protein expression is upregulated in glioma cells in response to treatment with Temozolomide (TMZ), the most common antiglioma chemotherapeutic agent, and that CHAC1 overexpression enhances glioma apoptotic death (Chen *et al*., 2017). CHAC1 acts as a pro‐apoptotic factor involved in apoptosis initiation and execution through the depletion of glutathione (Kumar *et al*., 2012; Mungrue *et al*., 2009). Moreover, TMZ‐induced upregulation of CHAC1 expression in glioma cells results in the binding of CHAC1 to the NOTCH3 protein and consequent inhibition of NOTCH3 activation, resulting in attenuation of NOTCH3‐mediated pathways (Chen *et al*., 2017). NOTCH activation has oncogenic roles in GBM (Lino *et al*., 2010; Sarkar *et al*., 2017; Takebe *et al*., 2014). Together, these observations suggest that mechanisms that negatively regulate *CHAC1* expression are involved in gliomagenesis.

**Figure 5:**
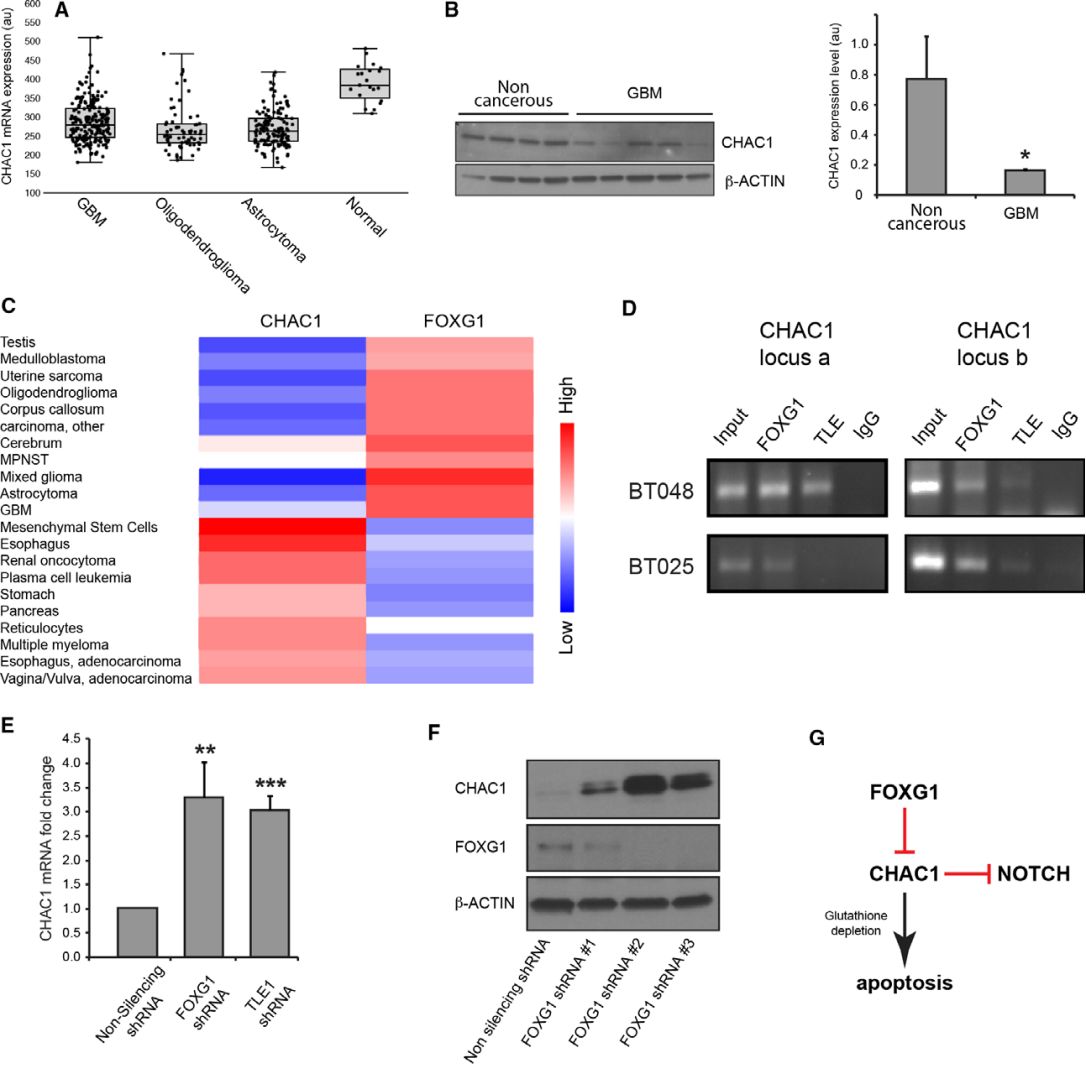
*CHAC1* is a FOXG1 target in BTICs. (A) *CHAC1* mRNA expression in normal brain and high‐ or low‐grade gliomas based on microarray data from Rembrandt database through the Betastasis portal. (B) Western blotting analysis showing the expression of CHAC1 protein in four noncancerous brain samples and five GBM samples (left panel). Average intensity of CHAC1 protein bands in noncancerous brain and GBM after normalization by β‐ACTIN expression (right panel, au = arbitrary units) (*P < 0.05). (C) Heatmap of mRNA expression of *CHAC1* and *FOXG1* in various normal and cancer samples. (D) ChIP analysis of FOXG1 and TLE1 binding to the *CHAC1* promoter at two different binding sites, operationally termed a and b. (E) Bar plot showing increase in *CHAC1* mRNA level following FOXG1 or TLE1 knockdown (*n* = 3; **P < 0.01; ***P < 0.001). (F) Western blotting analysis showing CHAC1 protein upregulation following FOXG1 knockdown using three different shRNA reagents. (G) Schematic model depicting a direct role for FOXG1 in the transcriptional repression of *CHAC1*, which is proposed to act both as pro‐apoptotic factor and inhibitor of NOTCH signalling in GBM cells.

We observed that both *CHAC1* mRNA and CHAC1 protein levels are lower in GBM compared to control samples from noncancerous brain tissues (Fig. 5A,B). More importantly, mRNA expression of *CHAC1* and *FOXG1* in selected samples from the MediSapiens database (Kilpinen *et al*., 2008) and The Cancer Genome Atlas showed an inverse correlation of *FOXG1* (high) and *CHAC1* (low) levels in GBM; in contrast, the opposite situation was observed in mesenchymal stem cells (Fig. 5C; Fig. S2). While both genes are tissue specific and are not expressed in most samples, tissues that do express these genes seem to preferentially express one or the other resulting in very few samples with high expression of both. Together, these results suggest that FOXG1 may repress *CHAC1* expression in GBM together with TLE1.

To examine this possibility further, we performed direct ChIP and RT‐qPCR experiments. Studies using two different BTIC lines showed that both FOXG1 and TLE1 are localized to the *CHAC1* promoter at two different genomic loci (Fig. 5D) (these loci are operationally termed ‘a’, located at chr15:41 237 139–41 237 414, and ‘b’, located at chr15:41 230 193–41 230 469 – Fig. S3). Control ChIP experiments using primers designed for negative control regions within the *CHAC1* locus revealed only negligible binding (Fig. S3). Consistent with these results, *CHAC1* mRNA increased in response to FOXG1 or TLE1 knockdown (Fig. 5E). CHAC1 protein was also upregulated following FOXG1 knockdown, as shown using three different *FOXG1*‐targeting shRNA sequences (Fig. 5F). Together, these results provide evidence suggesting that *CHAC1* is a direct FOXG1:TLE1 transcription repression target in GBM. Furthermore, they suggest that FOXG1:TLE1 may promote gliomagenesis, at least in part, through inhibition of the pro‐apoptotic and/or NOTCH inhibitory functions of CHAC1 (Fig. 5G).

## 4 Discussion

We utilized GBM patient‐derived cell cultures with *in vitro* stem‐like properties and *in vivo* tumour‐initiating ability to characterize FOXG1 and TLE1 genomewide occupancy patterns and identify their direct target genes. Combined ChIP‐seq and RNA‐seq studies, with the latter performed in BTICs with endogenous *vs* attenuated levels of FOXG1:TLE1, identified a subset of 150 genes as direct targets of FOXG1‐containing transcription repression complexes in GBM cells. Most of these direct FOXG1 targets showed increased expression following FOXG1 or TLE1 knockdown, in agreement with the demonstration that FOXG1 mediates transcriptional repression together with TLE proteins. Numerous FOXG1 peaks were not shared with TLE1, possibly because the efficiency of TLE1 immunoprecipitation was inferior to that of FOXG1 in ChIP experiments. This situation may also result in part from the broad participation of TLE1 in gene regulatory mechanisms with a variety of other transcription factors that recruit TLE proteins to DNA, thereby targeting TLE1 to many DNA sites not occupied by FOXG1.

As predicted based on previous studies showing promotion of GBM propagation by FOXG1:TLE1 (Verginelli *et al*., 2013), the identified target genes include several tumour suppressor, such as *APITD1*,* BRCA1* and *GADD45A* (Asuthkar *et al*., 2011; Krona *et al*., 2004; Silver and Livingston, 2012). We also identified as targets of FOXG1:TLE1 transcriptional repression a number of genes previously proposed to promote glioma invasion, including *EGR1*,* EGR2*,* GDF15*,* SERPINE1* and *SRPX2* (Codó *et al*., 2016; Han *et al*., 2014; Tang *et al*., 2016; Yukinaga *et al*., 2014). This finding, combined with the previous demonstration that FOXG1 promotes cancer stem‐like cell maintenance in GBM (Verginelli *et al*., 2013), raises the possibility that FOXG1:TLE1‐mediated transcription repression mechanisms may act to prevent transition of GBM stem‐like cells towards a more differentiated, ‘glioblast‐like’, state associated with enhanced migratory behaviour. This possibility is consistent with the involvement of FOXG1 and TLE1 in repressing the expression of genes associated with a more developmentally advanced astroglial phenotype, such as *GFAP*,* S100*β and GLUL (*glutamine synthetase)* (Verginelli *et al*., 2013).

Further insight into the mechanisms underlying the functions of FOXG1 and TLE1 complexes in GBM is provided by the present identification of *CHAC1* as a direct FOXG1:TLE1 target gene. CHAC1 expression is lower in GBM compared to control noncancerous brain (this study and Chen *et al*., 2017). Treatment of glioma cells with TMZ causes upregulation of *CHAC1* expression, which is in turn correlated with enhanced apoptotic cell death via caspase 3/9 activation (Chen *et al*., 2017). This response is consistent with the previously demonstrated role of CHAC1 as a pro‐apoptotic factor (Kumar *et al*., 2012; Mungrue *et al.,* 2009). When considered together with the previously shown prosurvival function of FOXG1 and TLE1 in healthy neurons (Dastidar *et al*., 2011, 2012), these observations suggest that FOXG1:TLE1 may promote glioma cell survival, at least in part, through inhibition of the pro‐apoptotic function of CHAC1 (Fig. 5G).

Another effect of TMZ‐induced upregulation of CHAC1 expression in glioma cells is the binding of CHAC1 to the NOTCH3 protein and consequent inhibition of NOTCH3 activation, resulting in attenuation of NOTCH3‐mediated pathways (Chen *et al*., 2017). NOTCH signalling has well‐recognized tumour‐promoting functions in GBM (Lino *et al*., 2010; Sarkar *et al*., 2017; Takebe *et al*., 2014). The ability of CHAC1 to inhibit NOTCH signalling was first recognized during murine cortical neurogenesis (Chi *et al*., 2012), when CHAC1 (referred to as Botch in that study) prevents maturation, and proper cell surface presentation, of NOTCH receptors by inhibiting the S1 furin‐like cleavage of the full‐length form of NOTCH. Based on these observations, it is reasonable to propose that FOXG1:TLE1 complexes cooperate with NOTCH signalling to promote gliomagenesis by directly repressing *CHAC1* expression in GBM cells (Fig. 5G).

The possibility that transcriptional programs controlled by FOXG1:TLE1 complexes may act to repress genes that negatively regulate NOTCH signalling in GBM, thereby contributing to maintenance of activated NOTCH pathways, is also consistent with the present identification of *GATA3* as another FOXG1:TLE1 transcription repression target in GBM. GATA3 was shown to induce human T‐cell lineage commitment in part by restraining NOTCH activity (Van de Walle *et al*., 2016). It is worth mentioning that our studies have identified *Delta and Notch‐like epidermal growth factor‐related receptor* (*DNER*) as an additional potential transcription repression target of FOXG1:TLE1. DNER inhibits GBM‐derived tumoursphere growth and promotes their differentiation *in vivo* and *in vitro*, opposite to the effect of FOXG1 and TLE1. Accordingly, DNER reduces the growth of brain tumour stem‐like cell‐initiated xenografts in host brains (Sun *et al*., 2009). DNER was proposed to act as an inhibitor of NOTCH signalling, although this possibility remains controversial (Eiraku *et al*., 2005; Greene *et al*., 2016). Together, these observations suggest that FOXG1 and NOTCH signalling pathways may functionally interact at various levels to promote gliomagenesis.

In conclusion, the identification of FOXG1:TLE1 target genes in GBM has provided evidence suggesting that this transcription repression complex promotes the tumourigenic potential of BTICs through a number of mechanisms impacting on several oncogenic pathways and has identified potentially attractive targets for antiglioma therapies.

## Author contributions

RD carried out experiments and helped draft the manuscript. FV carried out experiments. AP and RS participated in the design of high‐throughput genomic studies and provided reagents. SS participated in the design and coordination of the study and helped draft the manuscript. All authors read and approved the final manuscript.

## Supporting information


**Fig. S1.** FOXG1 binds FOXA1 DNA‐binding sites.
**Fig. S2.** CHAC1 gene expression as function of FOXG1 expression in GBM patients.
**Fig. S3.** FOXG1 binding sites upstream of CHAC1 gene.
**Table S1.** PCR and qPCR primers.
**Table S2.** Common genes differentially regulated in brain tumor‐initiating cells following FOXG1 or TLE1 knockdown.
**Table S3.** Gene ontology statistics.
**Table S4.** FOXG1‐regulated genes in brain tumor‐initiating cells.
**Table S5.** Common genes that are differentially regulated in brain tumor‐initiating cells following FOXG1 or TLE1 knockdown and have proximal FOXG1 binding sites.
