# Supplementary material for: Characterization of a FOXG1:TLE1 transcriptional network in glioblastoma‐initiating cells
Source: Mol Oncol. 2018 Apr 27;12(6):775–87. doi: 10.1002/1878-0261.12168 (PMC5983107; doi:10.1002/1878-0261.12168)
Supplement: Supplementary file 1 — Fig. S1. FOXG1 binds FOXA1 DNA‐binding sites. Fig. S2. CHAC1 gene expression as function of FOXG1 expression in GBM patients. Fig. S3. FOXG1 binding sites upstream of CHAC1 gene. Table S1. PCR and qPCR primers. Table S2. Common genes differentially regulated in brain tumor‐initiating cells following FOXG1 or TLE1 knockdown. Table S3. Gene ontology statistics. Table S4. FOXG1‐regulated genes in brain tumor‐initiating cells. Table S5. Common genes that are differentially regulated in brain tumor‐initiating cells following FOXG1 or TLE1 knockdown and have proximal FOXG1 binding sites. [file MOL2-12-775-s001.pdf]

SUPPLEMENTAL INFORMATION

Characterization of a FOXG1:TLE1 transcriptional network in glioblastoma initiating cells

Rola Dali[1,2], Federica Verginelli[1,3], Albena Pramatarova[4], Robert Sladek[4], Stefano Stifani[1,5]

[1]Department of Neurology and Neurosurgery, Montreal Neurological Institute, McGill University, Montreal, Canada;

[2]McGill Center for Bioinformatics, McGill University, Montreal, Canada;

[3] Current address: Laboratory of Cancer Stem Cell Research, Candiolo Cancer Institute, FPO-IRCCS, Candiolo, Italy;

[4]Departments of Human Genetics and Medicine, McGill University, Montreal, Canada.

[5]Corresponding author: E-mail, stefano.stifani@mcgill.ca.

A

```
FOXG1 MLDMGDRKEVKMIPKSSFSINSLVPEAVQNDNHHASHGHHNSHHPQHHHHHHHHHHHPPP
FOXA1 MLG-----TVKMEGHETSDWNSYYADTQEAYSSVPVSNMNSGLGSMNSMNTYMTMNTMTT
      **.      ***  :.: . **  .:: :  .  .  . :..  . :  : :   :  ..

FOXG1 PAPQPPPPPQQQQPPPPPPPPAPQPPQTRGAPAADDDKGPQQLLLPPPPPPPPAAALDGAK
FOXA1 SGNMTPASFNMSYANPGLGAGLSPGAVAGMPGG------------------SAGAMNSMT
      ..  .*.. : . . *   .. .*  . * *..                  .*.*::. .

FOXG1 ADGLGGKGEPGGGPGELAPVGPDEKEKGAGAG-------------GEEKKGAGEGGKDGE
FOXA1 AAGVTAMGT-ALSPSGMGAMGAQQAASMNGLGPYAAAMNPCMSPMAYAPSNLGRSRAGGG
      * *: . *  . .*. :...:*.::  .  * *             .   .. * .  .*

FOXG1 GGKEGEKKNGKYEKPPFSYNALIMMAIRQSPEKRLTLNGIYEFIMKNFPYYRENKQGWQN
FOXA1 GDAKTFKRSYPHAKPPYSYISLITMAIQQAPSKMLTLSEIYQWIMDLFPYYRQNQQRWQN
      *. :  *..  : ***:** :** ***.*:*.* ***. **::**. *****:*:* ***

FOXG1 SIRHNLSLNKCFVKVPRHYDDPGKGNYWMLDPSSDDVFIGGTTGKLRRRSTTSRAKLAFK
FOXA1 SIRHSLSFNDCFVKVARSPDKPGKGSYWTLHPDSGNMFENGCY--LRRQKRFKCEKQPGA
      ****.**:*.*****.*  *.****.** * *.*.::* .*    ***..  .  * .

FOXG1 RGARLTSTGLTFMDRAGSLYWPMSPFLSLHHPRASSTL--SYNGTTSAYPSHPMPYSSVL
FOXA1 GGGGGSGSGGS--GAKGGPESRKDPS-GASNPSADSPLHRGVHGKTGQLEGAPAPGPAAS
       *.  :.:* :  .  *.     .*  .  :* *.*.*  . :*.*.   . * * .:.

FOXG1 TQNSLGNNHSFSTANGLSVDRLVNGEIPYATHHLTAAALAASVPCGL-SVPCSG-TYSLN
FOXA1 PQTL---DHSGATATG--------GASELKTPASSTAPPISSGPGALASVPASHPAHGLA
      .*.    :** :**.*        *     *   ::*.  :* * .* ***.*  ::.*

FOXG1 PCSVNL-LAGQTSYFFPHVPHPSMTSQSSTSMSARAASSSTSPQAPSTLPCESLRP-SLP
FOXA1 PHESQLHLKGDPHYSFNH-PF-SINNLMSSSEQQHKLDFKAYEQALQYSPYGSTLPASLP
      * . :* * *:. * * * *. *:..  *:* . .  . .:  ** .  *  *  * ***

FOXG1 ----SFTTGLSGGLSDYFTHQNQGSSSNPLIH--
FOXA1 LGSASVTTRSPIEPSALEPAYYQGVYSRPVLNTS
          *.**  .   *   .   **  *.*:::
```

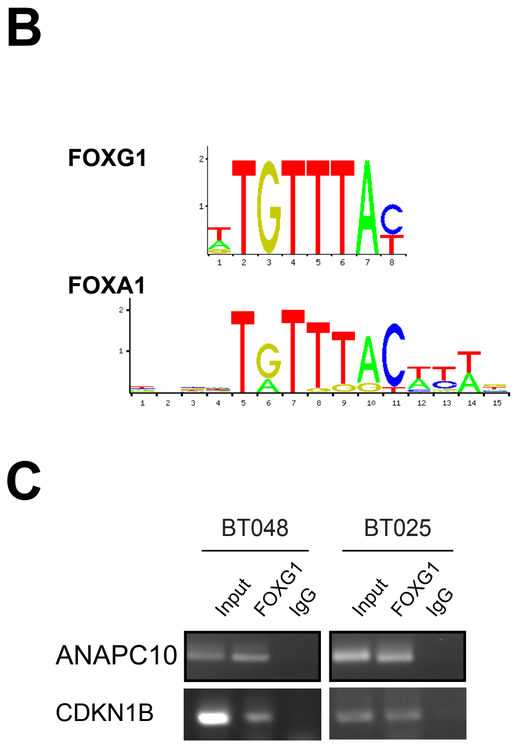

Fig. S1. FOXG1 binds FOXA1 DNA-binding sites. (A) Alignment of human FOXG1 and FOXA1 protein sequence. The conserved forkhead DNA-binding domain is highlighted in red font type. (B) Positional weight matrix of FOXG1 (top panel) and FOXA1 (bottom panel) based on JASPAR. (C) ChIP analysis of FOXG1 occupancy of FOXA1 identified binding sites from ENCODE using either anti-FOXG1 antibody or control rabbit immunoglobulin (IgG).

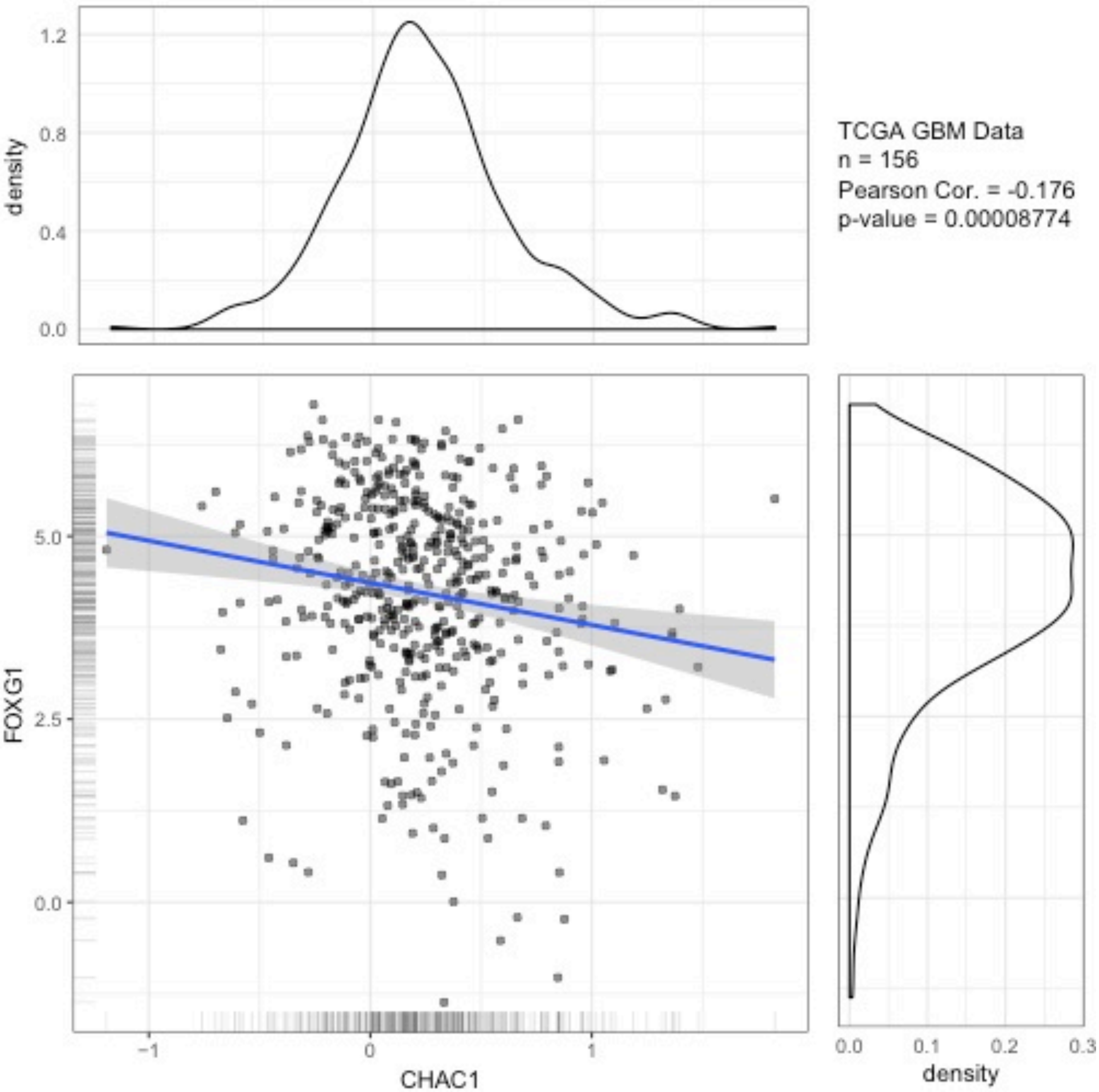

Fig. S2. CHAC1 gene expression as function of FOXG1 expression in GBM patients. Expression level of FOXG1 and CHAC1 was plotted in 156 GBM patients from the TCGA dataset (http://gliovis.bioinfo.cnio.es/ Agilent 4502A). FOXG1 and CHAC1 exhibit a negative correlation in GBM patients.

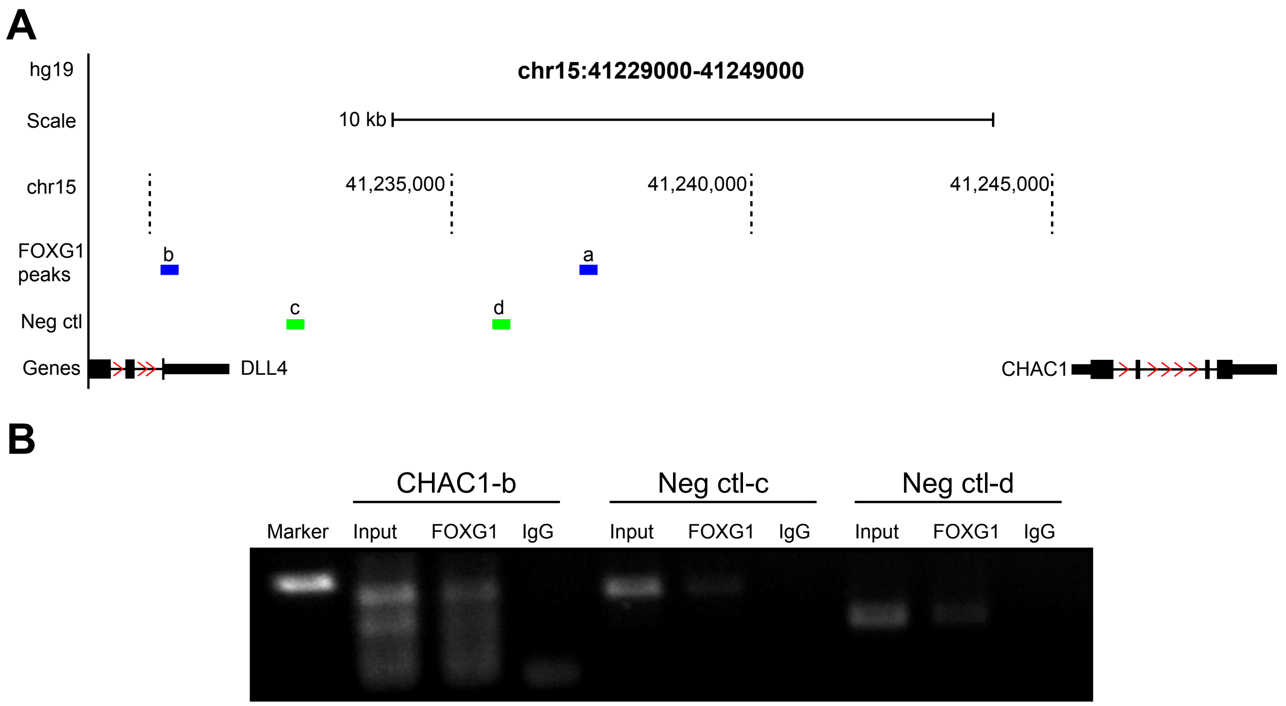

Fig. S3. FOXG1 binding sites upstream of *CHAC1* gene. (A) Two validated FOXG1 binding sites, annotated 'a' and 'b', are represented in blue rectangles). Sites 'c' and 'd' (green rectangles) correspond to negative control regions within the *CHAC1* locus. Plotted region represents chr15:41229000-41249000 of hg19. (B) FOXG1 ChIP analysis using either *CHAC1* primers for predicted binding site 'b' or negative control primers for sites 'c' and 'd' showing enrichment of FOXG1 ChIP as compared to IgG ChIP in BTIC line BT025.

Table S1: PCR and qPCR primers.

ChIP PCR Primer Sequences		
**Gene**	**Forward Sequence**	**Reverse Sequence**
ANAPC10	CTGAATCTGTAAATAGCTTTGGGC	GGGAAATGAGAATTACAAGATACTACTC
APITD1	GCCTTAGTGCAGAGATAGCAG	TGGGTGGAGAATGTTAAGTGC
BRCA1-a	GAAAAGCTTGTGGACAGTAACC	AGGGTGAATGGCAAGAGAAG
BRCA1-b	CTCAAGCTGACAAGAAAACATACAG	AGCTAAGATCATGCCACTGC
CHAC1-a	AGGTCCTCTTCGCACAAGA	TTATAGGTGTGAGCCATCGT
CHAC1-b	GTAACCTGTTTCCCTGCCT	CTTCTGTTCCTCTGGTTGG
CHAC1_Neg-c	CCCTCTCCTTTGCTGCTTAT	CTGTAGACTCACTTGGCTTCTT
CHAC1_Neg-d	TCCTCTAAGGCACAGGATAGT	GTGTGTCTGTGTCAAGGAGAG
CHRD	GTAGGTCTGTCAACATGAGTGG	AGGGCTTAGCAAACACAGTAG
DENND2D	AATCCCTTACCAAAAGCCAG	AGCTCCAGAGACTTATCCAA
DNER	GGTTGGAGCTGAATAAACGTTC	TTCTGCTCATATGGCCCATG
EGR1	GAAGAGAAGTGCTGCCTCT	TAGAGACAGAAAGACAGCCA
GADD45A	GTAAAGTCCATGAGGTTAGG	AAGAACTCAGAGAAGAGCCA
GATA3	CTCAGAAACAAGGATGTGGT	CCTTGTGCTGTTTCTTTGCA
GFAP	AACAGCCAGGACTCACTTG	GACTTCCATCTCCACTTCCTTG
GLB1	TCTGTTCTAACAGGGCTCTA	AACACTTTGAGAGGCTGAAG
GLUL	AGCCCAGAAATGTAAGACCC	AACACCCTAGACCTCCCTTAG
GPR37 -a	GACAAAGCAAACTGAGCCAAG	CGGGATAGCAATAAACGACCTC
GPR37 -b	TGAACATAGTGTCACAGGCC	GTGGTAGGTGGACTTTGCTC
KITLG	GCTAACTGCTACTGAATTGC	CTTCTTCACACCTGCCATT
NAV3	TTCGGCTTTCCTTCCCTATTG	CCTTACCGCTTCAGTGTTCTT
p21	GGAGTCAGATTCTGTGTGTG	CCTCTGCTTTCAGGCATTTC
p27	TCTGATCTCTACGCTCCCTG	CGAAAAGTAAGCAAGTCACAGTC
PCNA	GAGACTAAGGCAAGAGGACTG	ACACAGGCACGATAGTTATGTC
PSMD	CTGAGGTCAGGAGTTCAACAC	CTCACCTGTTTCCCCTGTTT
RB1	CAGTTTAACAAAGTCACTGGCG	GGTCATTCCAAAATAAGGATACCAC
RUNX1	TGGGCACCTACATATCATTCAC	CTAGTTCCCCTTTACCCACC
RUNX2	GGAAGTAGGTTTTGGAAGATAGGG	TCAGAACTGGAAAGCATCAGG
S100β	TTCCAAAGTCCATGCTCCC	CTCTTCACACTCCAATCTCCAC
SERPINE1	GTGAGCAGAAATCGCATCG	TGGTTTTCCGGGTTGAATCA
SOX2	TGTATTCTTGGCACCCTCATC	GTAACCCCAAAGACAATGAATAGC
TERT	ACTTTCAAGGCTCATTCCCC	CTTAATGCGTGAACCAGCTG
TRIM67	TTCTGATGTGAAGATGGGAT	AGGCTCTTCGATTTACCATG

q-PCR Primer Sequences		
**Gene**	**Forward Sequence**	**Reverse Sequence**
β-ACTIN	ATTGGCAATGAGCGGTTC	TGAAGGTAGTTTCGTGGATGC
BRCA1	AATAGAAACTACCCATCTCAAGAGG	GATTCCAGATTCCAGGTAAGGG
CHAC1	ACCATGTTGCCCTGCTTC	TGATGTCCACATGAGCACTC
DENND2A	TGACACGCACTTTATCGGAG	GGTTTGGACAATGACTGCTTG
DNER	CGAAAACAGGGCAGAAAGTTG	TCCAGAGCAAAATCAGTGAGG
EGR1	CAGCACCTTCAACCCTCAG	AGTCGAGTGGTTTGGCTG
FOXG1	CCTGCCCTGTGAGTCTTTAAG	GTTCACTTACAGTCTGGTCCC
GADD45A	GGGAAAGTCGCTACATGGATC	GTGTAGGGAGTAACTGCTTGAG
GATA3	GCGGGCTCTATCACAAAATG	TCCCCATTGGCATTCCTC
GFAP	ACCCAGCAACTCCAACTAAC	TCCTCATTCTAACGCAAGCTG
GLB1	CGGCCTTTTATATGGGGAAC	TAGCGGCCAAGGTTAAAGC
GLUL	TCAAGCAGGTGTACATGTCC	CGAAATTCCACTCAGGCAAC
KitLG	CCAGAACAGCTAAACGGAGTC	GACGAGAGGATTAAATAGGAGCAG
p21	TGTCACTGTCTTGTACCCTTG	GGCGTTTGGAGTGGTAGAA
RB1	CAGTTAATGCTATGTGTCCTTGAC	CTATCCGTGCACTCCTGTTC
RUNX1	CCAGGTTGCAAGATTTAATGACC	TTTTGATGGCTCTGTGGTAGG
RUNX2	AGCAAGGTTCAACGATCTGAG	GGCGGTCAGAGAACAAACTAG
S100β	CAGCAAGGAGACCAGGAAG	TGGAAAACGTCGATGAGGG
SERPINE1	AAGGCACCTCTGAGAACTTCA	CCCAGGACTAGGCAGGTG
SOX2	GAGAAGTTTGAGCCCCAGG	AGAGGCAAACTGGAATCAGG
TERT	CTCCATCCTGAAAGCCAAGAA	AGTCAGCTTGAGCAGGAATG
TRIM67	ACAAGGCACAACTATCTCAGG	ATCACACTGAGCAACGAGG

Table S2. Common genes differentially regulated in brain tumor-initiating cells following FOXG1 or TLE1 knockdown.
Genes that are differentially expressed following FOXG1 or TLE1 knockdown are listed based on RNA-seq data.

Gene	baseMean Scr	baseMean FOXG1	foldChange FOXG1	pval FOXG1	padj FOXG1	baseMean Scr	baseMean TLE1	foldChange TLE1	pval TLE1	padj TLE1
CHAC1	14.54	89.85	6.18	4.56E-05	0.013198583	14.54	96.44	6.63	2.33E-05	0.00319307
MIR30A	2597.74	8668.92	3.34	0.000512779	0.05973878	2597.74	10223.14	3.94	8.91E-05	0.007812905
BMF	13.52	40.91	3.03	0.000746221	0.073821636	13.52	47.22	3.49	0.000115329	0.009364181
MCHR1	391.91	1181.69	3.02	7.56E-14	6.69E-10	391.91	918.83	2.34	6.71E-09	6.25E-06
STX3	27.02	81.33	3.01	5.47E-06	0.002985839	27.02	73.06	2.70	4.73E-05	0.005159577
NEK10	38.06	108.34	2.85	4.60E-05	0.013198583	38.06	109.82	2.89	3.61E-05	0.004229725
SLC7A11	327.07	925.26	2.83	0.000616893	0.066673589	327.07	1433.85	4.38	1.76E-06	0.000451868
AHNAK2	53.53	147.74	2.76	2.57E-08	5.06E-05	53.53	135.61	2.53	3.90E-07	0.000150273
SERPINE1	1067.51	2840.23	2.66	5.70E-05	0.015337932	1067.51	2497.33	2.34	0.000452403	0.022440212
C2orf66	102.61	263.48	2.57	1.35E-05	0.005545299	102.61	307.13	2.99	4.30E-07	0.000158472
GDF15	715.63	1835.51	2.56	1.51E-07	0.000243139	715.63	1690.13	2.36	1.59E-06	0.000420401
SDSL	41.54	103.83	2.5	1.16E-05	0.005143102	41.54	98.39	2.37	3.92E-05	0.004508124
GPNMB	53.53	127.7	2.39	5.56E-06	0.002985839	53.53	106.83	2.00	0.000369995	0.019499593
ADAMTSL4	91.11	214.7	2.36	0.000402128	0.050978276	91.11	179.38	1.97	0.005226706	0.09611061
ABCA12	111.09	257.04	2.31	1.05E-05	0.004916185	111.09	319.05	2.87	2.85E-08	1.94E-05
CDH13	65.51	146.66	2.24	1.13E-05	0.005132214	65.51	121.70	1.86	0.000866615	0.033506582
HIST1H2BD	42.03	92.34	2.2	0.00024091	0.040428813	42.03	93.43	2.22	0.000191107	0.013375955
PLA2G4C	93.07	204.14	2.19	3.74E-06	0.002549094	93.07	249.47	2.68	4.98E-09	4.90E-06
CHRNA9	155.63	335.27	2.15	5.17E-09	1.52E-05	155.63	352.79	2.27	4.39E-10	6.47E-07
SPOCD1	613.72	1297.78	2.11	0.000361632	0.048637899	613.72	1186.71	1.93	0.001664079	0.049608599
BLVRB	68.57	141.23	2.06	6.82E-05	0.017506878	68.57	123.25	1.80	0.001378162	0.044131089
RBM47	180.59	368.6	2.04	4.44E-05	0.013198583	180.59	378.05	2.09	2.37E-05	0.00320739
NR4A3	161.12	326.05	2.02	0.000130266	0.025950843	161.12	302.07	1.87	0.000647835	0.028255838
RENBP	196.66	390.34	1.98	0.000563743	0.063264214	196.66	387.15	1.97	0.000657149	0.028451813
LOC100126784	117.09	228.04	1.95	2.64E-05	0.009291451	117.09	296.67	2.53	3.33E-09	3.47E-06
APLP1	1726.58	3214.75	1.86	5.13E-09	1.52E-05	1726.58	3467.85	2.01	6.00E-11	1.06E-07
HAP1	732.94	1363.12	1.86	3.45E-13	2.04E-09	732.94	1705.70	2.33	3.64E-23	6.44E-19
MARCH3	78.04	144.19	1.85	0.00038701	0.050514637	78.04	132.18	1.69	0.002499713	0.062521072
CACNG5	103.59	188.2	1.82	0.000253996	0.040707863	103.59	196.28	1.89	8.61E-05	0.007677114
ZNF436	2692.71	4895.41	1.82	9.75E-16	1.73E-11	2692.71	5072.96	1.88	1.80E-17	6.38E-14
KCNB1	141.58	250.03	1.77	2.62E-05	0.009291451	141.58	281.72	1.99	3.02E-07	0.000121574
RGS16	1664	2937.99	1.77	0.000118232	0.024924471	1664.00	2684.33	1.61	0.001182802	0.040512671
BEST1	104.07	182.68	1.76	0.000245534	0.040428813	104.07	194.80	1.87	4.06E-05	0.004606296
MAP6	253.62	446.14	1.76	1.67E-07	0.000246822	253.62	721.46	2.84	7.98E-23	7.06E-19
CNIH3	853.41	1490.56	1.75	1.26E-05	0.00530154	853.41	1438.34	1.69	4.39E-05	0.004918974
ELL2	96.07	166.21	1.73	0.000673421	0.070146688	96.07	154.53	1.61	0.003363523	0.074326096
LONRF2	1884.4	3258.18	1.73	0.000823637	0.078370158	1884.40	4429.79	2.35	2.21E-07	9.33E-05
GGN	113.56	194.07	1.71	0.000344505	0.047290666	113.56	198.27	1.75	0.000195044	0.013391976
SCG2	6661.68	11233.62	1.69	0.000528111	0.061122783	6661.68	10964.27	1.65	0.000955228	0.035420515
RND3	1007.73	1689.61	1.68	1.34E-08	3.39E-05	1007.73	1319.61	1.31	0.003104573	0.070753894
AKAP6	653.29	1092.94	1.67	1.69E-06	0.001428904	653.29	1824.34	2.79	1.78E-21	7.88E-18
SERTAD1	319.73	533.47	1.67	1.38E-06	0.001287708	319.73	498.40	1.56	3.00E-05	0.003786244
BHLHE41	271.61	450.58	1.66	4.94E-06	0.002823613	271.61	456.99	1.68	2.61E-06	0.000600948
C10orf55	245.6	408.62	1.66	1.60E-05	0.006166306	245.60	405.47	1.65	2.19E-05	0.003078942
ZNF436-AS1	544.46	901.33	1.66	3.21E-05	0.01051956	544.46	932.69	1.71	8.97E-06	0.001573113
FOSL2	1187.44	1956.13	1.65	2.62E-06	0.001853834	1187.44	1842.90	1.55	3.49E-05	0.004115324
KLF5	215.21	354.4	1.65	0.000431321	0.052982015	215.21	354.25	1.65	0.000433399	0.021741171
KANK3	179.13	294.49	1.64	0.000147929	0.027462933	179.13	312.56	1.74	2.01E-05	0.002915754
AZIN2	144.61	235.57	1.63	0.00043336	0.052982015	144.61	227.56	1.57	0.001103924	0.038480887
METTL7B	2120.98	3463.5	1.63	1.83E-08	4.04E-05	2120.98	3105.10	1.46	1.21E-05	0.002065133
NUAK2	286.24	467.09	1.63	8.68E-06	0.004424398	286.24	428.75	1.50	0.000256617	0.015615703
GPR37	171.14	273.48	1.6	0.000362559	0.048637899	171.14	248.96	1.45	0.004619137	0.089137846
SLC35D2	720.86	1143.73	1.59	0.000661458	0.069431001	720.86	1055.54	1.46	0.004949168	0.093245372
HBEGF	291.73	462.07	1.58	0.000327627	0.046044538	291.73	483.01	1.66	8.09E-05	0.007528558
SERPINB8	463.36	733.82	1.58	9.49E-05	0.022703107	463.36	736.78	1.59	8.12E-05	0.007528558
ZNF671	334.28	525.08	1.57	9.23E-05	0.022378793	334.28	541.10	1.62	2.97E-05	0.003786244
PLAU	719.89	1118.1	1.55	4.53E-06	0.002802236	719.89	1185.56	1.65	2.03E-07	9.00E-05
ABTB2	1116.78	1714.33	1.54	0.000299091	0.043184528	1116.78	2295.23	2.06	1.37E-09	1.61E-06
NRP2	15538.21	23851.78	1.54	2.22E-06	0.001710787	15538.21	28186.21	1.81	5.74E-11	1.06E-07
TMEM59L	543.86	837.32	1.54	0.00073466	0.073821636	543.86	805.54	1.48	0.002140928	0.05735484
NEDD9	2681.22	4096.57	1.53	0.000940917	0.084150337	2681.22	4052.58	1.51	0.001274312	0.042021445

HID1	323.16	492.49	1.52	7.97E-05	0.020162684	323.16	504.36	1.56	3.04E-05	0.003787628
RIN1	657.89	999.63	1.52	8.74E-06	0.004424398	657.89	1131.94	1.72	7.50E-09	6.64E-06
SOCS3	616.98	937.16	1.52	4.75E-06	0.002802236	616.98	851.06	1.38	0.000446834	0.022226221
TCN2	421.26	640.85	1.52	2.54E-05	0.009291451	421.26	628.56	1.49	5.95E-05	0.006092489
PROB1	201.14	303.01	1.51	0.000987707	0.087016521	201.14	319.49	1.59	0.00018812	0.013324891
FAM84B	3003.88	4495.46	1.5	4.24E-05	0.013198583	3003.88	4964.82	1.65	3.42E-07	0.000134541
HIVEP3	1825.03	2745.84	1.5	0.000149967	0.027462933	1825.03	2971.73	1.63	6.25E-06	0.001190157
LOC100652768	332.66	497.58	1.5	0.000156904	0.028065209	332.66	494.88	1.49	0.000192833	0.013391976
SH3BP5-AS1	997.56	1496.73	1.5	0.000452374	0.054494133	997.56	1546.45	1.55	0.000151345	0.011404343
SLC22A4	293.67	440.09	1.5	0.000256418	0.040707863	293.67	424.74	1.45	0.000871901	0.03353596
IRS2	11522.84	17201.82	1.49	0.000305764	0.043418537	11522.84	17411.19	1.51	0.000202974	0.013655425
LRRN2	706.3	1047.46	1.48	1.02E-05	0.004916185	706.30	924.07	1.31	0.002717613	0.065385168
ORAI3	442.73	655.27	1.48	0.000150435	0.027462933	442.73	624.64	1.41	0.000899326	0.034064381
SERPINE2	4959.64	7364.86	1.48	4.89E-08	8.66E-05	4959.64	6423.04	1.30	0.000359478	0.019232622
FGF1	366.23	535.35	1.46	0.000198417	0.034446793	366.23	703.03	1.92	1.11E-10	1.78E-07
OSBP2	2632.61	3836.66	1.46	5.59E-07	0.00061824	2632.61	3317.62	1.26	0.002127249	0.057161337
EFNA1	517.37	750.19	1.45	0.000103573	0.022703107	517.37	683.61	1.32	0.003702002	0.077902514
ARL4C	5485.66	7889.27	1.44	4.77E-07	0.000589922	5485.66	7342.02	1.34	5.27E-05	0.005627022
NAP1L3	373.68	533.54	1.43	0.000564476	0.063264214	373.68	584.23	1.56	1.38E-05	0.002282803
RNF19A	11009.86	15705.36	1.43	5.00E-07	0.000589922	11009.86	16462.40	1.50	1.28E-08	9.84E-06
GRIK2	666.81	947.23	1.42	0.000103368	0.022703107	666.81	925.90	1.39	0.000282306	0.016775444
KCNF1	7027.1	9992.74	1.42	3.17E-05	0.01051956	7027.10	10902.15	1.55	2.20E-07	9.33E-05
CHST2	2181.18	3081.72	1.41	6.45E-05	0.016790371	2181.18	3088.63	1.42	5.85E-05	0.006045509
COL4A1	42223.41	59635.59	1.41	6.98E-07	0.000727465	42223.41	61367.97	1.45	7.98E-08	4.05E-05
NAP1L5	559.27	788.51	1.41	0.000246573	0.040428813	559.27	824.77	1.47	3.29E-05	0.003952858
PROX1	4566.77	6422.14	1.41	1.03E-05	0.004916185	4566.77	6727.90	1.47	5.50E-07	0.000194715
ALK	4251.55	5958.95	1.4	0.000662629	0.069431001	4251.55	8171.88	1.92	5.59E-11	1.06E-07
CRIM1	3346.2	4701.23	1.4	4.30E-06	0.002802236	3346.20	4493.95	1.34	6.73E-05	0.006586516
MAPK10	1041.97	1461.6	1.4	4.62E-05	0.013198583	1041.97	1637.47	1.57	5.00E-08	2.85E-05
MATN2	9003.74	12624.2	1.4	1.97E-06	0.001584194	9003.74	12449.32	1.38	5.09E-06	0.00102353
NOS1AP	659.42	914.69	1.39	0.000296037	0.043184528	659.42	979.74	1.49	1.15E-05	0.001996652
SNPH	941.45	1309.6	1.39	9.71E-05	0.022703107	941.45	1396.28	1.48	3.15E-06	0.000706902
SYTL5	454.3	632.62	1.39	0.000807758	0.077729359	454.30	633.00	1.39	0.000798608	0.031779206
MARCH2	489.71	674.73	1.38	0.000975197	0.086343952	489.71	688.64	1.41	0.000446757	0.022226221
PLCD3	2795.86	3872.18	1.38	1.49E-05	0.005875284	2795.86	4060.50	1.45	7.06E-07	0.000223206
SLC9A7	695.93	960.26	1.38	0.000331703	0.046108929	695.93	990.24	1.42	8.27E-05	0.007629713
AMIGO2	596.43	819.23	1.37	0.000554883	0.062986364	596.43	974.75	1.63	8.01E-08	4.05E-05
C1orf198	2595.74	3562.67	1.37	2.68E-05	0.009291451	2595.74	3300.64	1.27	0.001433663	0.045445615
C1orf226	1067.68	1461.68	1.37	0.000152663	0.027585204	1067.68	1614.61	1.51	5.88E-07	0.000204167
DNMBP-AS1	759.4	1037.97	1.37	0.000402713	0.050978276	759.40	1051.71	1.38	0.000224965	0.014690656
GPC1	25272.55	34307.99	1.36	1.20E-05	0.005168069	25272.55	32471.50	1.28	0.000331065	0.018263249
MAN2B2	2372.31	3214.72	1.36	6.19E-05	0.016367731	2372.31	3232.16	1.36	4.60E-05	0.005125234
P4HTM	1031.49	1399.41	1.36	0.000268874	0.041361015	1031.49	1449.92	1.41	4.71E-05	0.005159577
PCNXL2	1839.24	2475.24	1.35	0.000403036	0.050978276	1839.24	3043.41	1.65	1.99E-09	2.20E-06
RGMA	1840.04	2478.46	1.35	0.000125448	0.025533716	1840.04	2539.39	1.38	3.34E-05	0.003965603
SHROOM2	2498.4	3374.53	1.35	8.63E-05	0.021297263	2498.40	3205.40	1.28	0.001139246	0.039325101
DUSP3	3569.15	4779.36	1.34	0.000923989	0.083907686	3569.15	4740.43	1.33	0.001284757	0.042287133
LAMB2	17914.23	23963.84	1.34	3.34E-05	0.010763391	17914.23	24464.70	1.37	8.93E-06	0.001573113
MBD5	1431.09	1918.57	1.34	0.00030649	0.043418537	1431.09	1868.52	1.31	0.001020112	0.036567081
PROS1	4485.99	6012.43	1.34	0.001136067	0.09534351	4485.99	5951.30	1.33	0.001693115	0.049886314
DDR1	2626.31	3500.26	1.33	0.000137989	0.026559959	2626.31	3403.24	1.30	0.000585914	0.026671877
FZD7	3825.23	5085.49	1.33	0.000105131	0.022703107	3825.23	5206.47	1.36	2.66E-05	0.003537087
ITPKC	1298.2	1720.46	1.33	0.000490814	0.057558562	1298.20	1954.01	1.51	4.02E-07	0.000151553
KALRN	2675.52	3564.68	1.33	0.000132753	0.025950843	2675.52	3888.92	1.45	6.34E-07	0.000204167
LRP10	5794.76	7699.77	1.33	0.000104939	0.022703107	5794.76	7956.69	1.37	1.52E-05	0.002409695
RDH10	1024.45	1359.87	1.33	0.000755847	0.074226372	1024.45	1360.33	1.33	0.00074548	0.030471468
CREBRF	832.62	1102.36	1.32	0.001197905	0.098206004	832.62	1249.52	1.50	2.61E-06	0.000600948
DKK3	11885.84	15635.32	1.32	0.00010248	0.022703107	11885.84	14582.02	1.23	0.00379349	0.0786594
DNMBP	5532.82	7300.37	1.32	0.000122043	0.02529017	5532.82	7233.75	1.31	0.000203446	0.013655425
GBE1	1011	1329.46	1.31	0.001169815	0.097265226	1011.00	1319.98	1.31	0.001568555	0.047807184
LYST	2644.46	3473.7	1.31	0.000286455	0.042342253	2644.46	3767.50	1.42	2.51E-06	0.000593317
PLEKHG1	2289.41	2995.1	1.31	0.000408861	0.051348327	2289.41	3480.40	1.52	3.48E-08	2.20E-05
RPS6KA2	12393.4	16280.65	1.31	0.000108736	0.02319886	12393.40	16757.85	1.35	1.90E-05	0.002798255
SMPD1	2232.58	2932.28	1.31	0.000359429	0.048637899	2232.58	2937.66	1.32	0.000330839	0.018263249
FRMD5	3102.85	4027.1	1.3	0.000461542	0.054507908	3102.85	3893.51	1.25	0.002312198	0.059598838
KIAA0513	1949.34	2538.14	1.3	0.000617488	0.066673589	1949.34	3028.64	1.55	1.08E-08	8.67E-06

NPTXR	6891.62	8944.57	1.3	0.000266677	0.041361015	6891.62	9516.11	1.38	6.58E-06	0.00123072
ENC1	11652.26	15041.36	1.29	0.00029996	0.043184528	11652.26	16918.97	1.45	1.30E-07	6.06E-05
GLIS3	3059.03	3932.08	1.29	0.000758695	0.074226372	3059.03	4012.57	1.31	0.000269983	0.016097192
NMNAT2	1972.33	2546.45	1.29	0.000933068	0.084150337	1972.33	2862.22	1.45	1.38E-06	0.000388186
EHD2	7049.05	9027.35	1.28	0.000547828	0.062717271	7049.05	8764.15	1.24	0.002349497	0.059818033
SHC1	4325.65	5525.43	1.28	0.000791225	0.076562888	4325.65	5822.52	1.35	4.67E-05	0.005159577
TPP1	7434.01	9485.46	1.28	0.000646177	0.068930734	7434.01	10001.79	1.35	3.30E-05	0.003952858
CX3CL1	4468.04	5691.91	1.27	0.000882155	0.081786385	4468.04	6422.94	1.44	6.23E-07	0.000204167
CNTNAP1	9990.41	12635.48	1.26	0.000913456	0.083378741	9990.41	13034.25	1.30	0.000176131	0.012730287
IDS	13878.13	17536.63	1.26	0.000880096	0.081786385	13878.13	19666.47	1.42	7.43E-07	0.000230818
EFS	8084.19	6366.97	0.79	0.000910997	0.083378741	8084.19	6119.20	0.76	0.000110588	0.00915089
FDFT1	18744.36	14714.47	0.79	0.000586954	0.064557683	18744.36	14121.53	0.75	5.86E-05	0.006045509
CCNF	5121.55	3954.64	0.77	0.000617382	0.066673589	5121.55	3949.08	0.77	0.00057854	0.026429794
HMGCS1	51072.73	39166.46	0.77	0.000972191	0.086343952	51072.73	37929.24	0.74	0.000221102	0.014554905
IDH1	19296.62	14933.56	0.77	0.000270944	0.041361015	19296.62	14713.51	0.76	0.00011742	0.009366132
NETO1	2270.59	1754.54	0.77	0.001049089	0.091966664	2270.59	1815.23	0.80	0.004451927	0.087110191
SUV39H2	2459.85	1885.28	0.77	0.000661854	0.069431001	2459.85	1954.62	0.79	0.003241133	0.072558766
MIS18A	1583.24	1196.59	0.76	0.000728083	0.073821636	1583.24	1185.68	0.75	0.000487261	0.023646437
HAUS1	1753.4	1299.28	0.74	0.00025747	0.040707863	1753.40	1173.27	0.67	1.05E-06	0.000310423
LOC100129518	1401.63	1012.48	0.72	0.000130751	0.025950843	1401.63	940.76	0.67	2.95E-06	0.000670812
POP1	2071.84	1483.7	0.72	3.03E-05	0.010333898	2071.84	1544.35	0.75	0.000239319	0.015244128
SKP2	4924.55	3568.77	0.72	4.49E-05	0.013198583	4924.55	3186.00	0.65	3.75E-08	2.21E-05
CETN3	869.93	616.78	0.71	0.000224744	0.038266928	869.93	639.90	0.74	0.000961254	0.035462247
FIGNL1	1220.49	870.78	0.71	0.000100709	0.022703107	1220.49	929.87	0.76	0.001689309	0.049886314
RMI2	1244.61	871.69	0.7	4.51E-05	0.013198583	1244.61	767.46	0.62	3.66E-08	2.21E-05
TNFAIP6	834.96	538.91	0.65	4.64E-06	0.002802236	834.96	526.55	0.63	1.45E-06	0.000400661
SNORD30	166.08	92.8	0.56	0.000705802	0.072244699	166.08	81.93	0.49	4.75E-05	0.005159577
PMCH	346.19	185.61	0.54	8.97E-07	0.000882036	346.19	217.05	0.63	0.000195873	0.013391976

Table S3: Gene Ontology Statistics.

	FOXG1					
GO biological process complete	Genes in Ontology	Genes in treatment	Expected	Fold Enrichment	+/-	P value
system development	4034	87	41.36	2.1	+	5.00E-09
regulation of cell communication	3067	64	31.44	2.04	+	9.10E-05
angiogenesis	301	17	3.09	5.51	+	1.73E-04
blood vessel morphogenesis	381	19	3.91	4.86	+	1.76E-04
nervous system development	2204	51	22.59	2.26	+	1.85E-04
regulation of signal transduction	2765	57	28.35	2.01	+	1.20E-03
positive regulation of locomotion	452	19	4.63	4.1	+	2.40E-03
cell differentiation	3290	63	33.73	1.87	+	2.98E-03
chemotaxis	483	18	4.95	3.64	+	2.67E-02
regulation of cell death	1545	36	15.84	2.27	+	2.80E-02
positive regulation of cell proliferation	871	25	8.93	2.8	+	3.39E-02
	TLE					
chromosome organization	543	83	25.09	3.31	+	9.07E-17
DNA conformation change	258	45	11.92	3.77	+	9.57E-10
developmental process	5325	342	246.04	1.39	+	4.26E-08
RNA splicing	377	51	17.42	2.93	+	2.37E-07
nervous system development	2204	166	101.83	1.63	+	2.45E-06
regulation of cell communication	3067	213	141.71	1.5	+	4.98E-06
DNA packaging	175	30	8.09	3.71	+	1.73E-05
regulation of signal transduction	2765	190	127.76	1.49	+	1.25E-04
regulation of cell death	1545	120	71.39	1.68	+	2.09E-04
regulation of apoptotic process	1431	113	66.12	1.71	+	2.41E-04
chromatin assembly	135	24	6.24	3.85	+	3.56E-04
histone exchange	46	13	2.13	6.12	+	3.20E-03
neurogenesis	1500	111	69.31	1.6	+	7.56E-03
regulation of synaptic plasticity	144	22	6.65	3.31	+	1.51E-02
cell differentiation	3290	206	152.01	1.36	+	2.35E-02
cell proliferation	696	60	32.16	1.87	+	4.04E-02

Table S4. FOXG1-regulated genes in brain tumor-initiating cells
FOXG1 ChIP-seq data were cross-analyzed with RNA-seq data. Genes that are differentially expressed in FOXG1-attenuated cells and have proximal FOXG1 binding sites are listed.

id	baseMean.Scr	baseMean.FOXG1	foldChange	log2FoldChang	pval	padj
KRT80	7.01	34.42	4.91	2.3	0.000146286	0.027462933
MIR30A	2597.74	8668.92	3.34	1.74	0.000512779	0.05973878
BMF	13.52	40.91	3.03	1.6	0.000746221	0.073821636
MCHR1	391.91	1181.69	3.02	1.59	7.56E-14	6.69E-10
STX3	27.02	81.33	3.01	1.59	0.00000547	0.002985839
NEK10	38.06	108.34	2.85	1.51	0.000046	0.013198583
SLC7A11	327.07	925.26	2.83	1.5	0.000616893	0.066673589
AHNAK2	53.53	147.74	2.76	1.46	2.57E-08	0.0000506
SERPINE1	1067.51	2840.23	2.66	1.41	0.000057	0.015337932
PCDHA8	23.01	59.88	2.6	1.38	0.000577985	0.0639685
GDF15	715.63	1835.51	2.56	1.36	0.000000151	0.000243139
GPNMB	53.53	127.7	2.39	1.25	0.00000556	0.002985839
ABCA12	111.09	257.04	2.31	1.21	0.0000105	0.004916185
CDH13	65.51	146.66	2.24	1.16	0.0000113	0.005132214
AQP3	54.03	119.27	2.21	1.14	0.0000572	0.015337932
HIST1H2BD	42.03	92.34	2.2	1.14	0.00024091	0.040428813
PLA2G4C	93.07	204.14	2.19	1.13	0.00000374	0.002549094
CHRNA9	155.63	335.27	2.15	1.11	5.17E-09	0.0000152
EGR2	563.4	1166.85	2.07	1.05	3.59E-10	0.00000159
BLVRB	68.57	141.23	2.06	1.04	0.0000682	0.017506878
RBM47	180.59	368.6	2.04	1.03	0.0000444	0.013198583
DMRTA1	57.04	116.24	2.04	1.03	0.00026375	0.041331741
NR4A3	161.12	326.05	2.02	1.02	0.000130266	0.025950843
SRPX2	259.69	486.4	1.87	0.91	0.000682788	0.070706532
HAP1	732.94	1363.12	1.86	0.9	3.45E-13	2.04E-09
MARCH3	78.04	144.19	1.85	0.89	0.00038701	0.050514637
KCNB1	141.58	250.03	1.77	0.82	0.0000262	0.009291451
RGS16	1664	2937.99	1.77	0.82	0.000118232	0.024924471
MAP6	253.62	446.14	1.76	0.81	0.000000167	0.000246822
BEST1	104.07	182.68	1.76	0.81	0.000245534	0.040428813
CNIH3	853.41	1490.56	1.75	0.8	0.0000126	0.00530154
ELL2	96.07	166.21	1.73	0.79	0.000673421	0.070146688
LONRF2	1884.4	3258.18	1.73	0.79	0.000823637	0.078370158
C10orf10	251.17	428.32	1.71	0.77	0.000180232	0.031599434
RND3	1007.73	1689.61	1.68	0.75	1.34E-08	0.0000339
AKAP6	653.29	1092.94	1.67	0.74	0.00000169	0.001428904
MAP3K14	815.67	1352.93	1.66	0.73	0.001109038	0.094873677
FOSL2	1187.44	1956.13	1.65	0.72	0.00000262	0.001853834
KANK3	179.13	294.49	1.64	0.72	0.000147929	0.027462933
KLF5	215.21	354.4	1.65	0.72	0.000431321	0.052982015
METTL7B	2120.98	3463.5	1.63	0.71	1.83E-08	0.0000404

NUAK2	286.24	467.09	1.63	0.71	0.00000868	0.004424398
MAFK	329.25	536.68	1.63	0.7	0.000839507	0.07907441
GPR37	171.14	273.48	1.6	0.68	0.000362559	0.048637899
SLC35D2	720.86	1143.73	1.59	0.67	0.000661458	0.069431001
CLU	13692.69	21841.04	1.6	0.67	0.001169951	0.097265226
ACTA2-AS1	428.75	677.38	1.58	0.66	0.000279023	0.041872304
NRP2	15538.21	23851.78	1.54	0.62	0.00000222	0.001710787
ABTB2	1116.78	1714.33	1.54	0.62	0.000299091	0.043184528
TMEM59L	543.86	837.32	1.54	0.62	0.00073466	0.073821636
HID1	323.16	492.49	1.52	0.61	0.0000797	0.020162684
NEDD9	2681.22	4096.57	1.53	0.61	0.000940917	0.084150337
RIN1	657.89	999.63	1.52	0.6	0.00000874	0.004424398
HIVEP3	1825.03	2745.84	1.5	0.59	0.000149967	0.027462933
FAM84B	3003.88	4495.46	1.5	0.58	0.0000424	0.013198583
MICAL2	1384.39	2064.82	1.49	0.58	0.0000866	0.021297263
SLC22A4	293.67	440.09	1.5	0.58	0.000256418	0.040707863
SERPINE2	4959.64	7364.86	1.48	0.57	4.89E-08	0.0000866
ORAI3	442.73	655.27	1.48	0.57	0.000150435	0.027462933
KLF4	313.73	463.18	1.48	0.56	0.00037327	0.04969825
FGF1	366.23	535.35	1.46	0.55	0.000198417	0.034446793
SLC5A9	326.68	479.63	1.47	0.55	0.000461723	0.054507908
FGFR2	2916.98	4250.68	1.46	0.54	0.00000049	0.000589922
OSBP2	2632.61	3836.66	1.46	0.54	0.000000559	0.00061824
EFNA1	517.37	750.19	1.45	0.54	0.000103573	0.022703107
RNF19A	11009.86	15705.36	1.43	0.51	0.0000005	0.000589922
GRIK2	666.81	947.23	1.42	0.51	0.000103368	0.022703107
COL4A1	42223.41	59635.59	1.41	0.5	0.000000698	0.000727465
UBTD1	1850.63	2622.64	1.42	0.5	0.000416217	0.051904053
MATN2	9003.74	12624.2	1.4	0.49	0.00000197	0.001584194
RPH3A	4652.77	6555.7	1.41	0.49	0.00000243	0.001792469
CRIM1	3346.2	4701.23	1.4	0.49	0.0000043	0.002802236
PROX1	4566.77	6422.14	1.41	0.49	0.0000103	0.004916185
MAPK10	1041.97	1461.6	1.4	0.49	0.0000462	0.013198583
ALK	4251.55	5958.95	1.4	0.49	0.000662629	0.069431001
SNPH	941.45	1309.6	1.39	0.48	0.0000971	0.022703107
SYTL5	454.3	632.62	1.39	0.48	0.000807758	0.077729359
PLCD3	2795.86	3872.18	1.38	0.47	0.0000149	0.005875284
NOS1AP	659.42	914.69	1.39	0.47	0.000296037	0.043184528
ARID5A	1091.73	1508.46	1.38	0.47	0.000333292	0.046108929
C1orf198	2595.74	3562.67	1.37	0.46	0.0000268	0.009291451
CXADR	1443.9	1991.73	1.38	0.46	0.00024527	0.040428813
SLC9A7	695.93	960.26	1.38	0.46	0.000331703	0.046108929
AMIGO2	596.43	819.23	1.37	0.46	0.000554883	0.062986364
SERTAD3	524.42	723.67	1.38	0.46	0.000859262	0.080506907
MARCH2	489.71	674.73	1.38	0.46	0.000975197	0.086343952

C1orf226	1067.68	1461.68	1.37	0.45	0.000152663	0.027585204
GM2A	1057.15	1448.98	1.37	0.45	0.000170687	0.030225181
TIGD6	952.17	1297.2	1.36	0.45	0.001083278	0.094495955
GPC1	25272.55	34307.99	1.36	0.44	0.000012	0.005168069
MAN2B2	2372.31	3214.72	1.36	0.44	0.0000619	0.016367731
P4HTM	1031.49	1399.41	1.36	0.44	0.000268874	0.041361015
SHROOM2	2498.4	3374.53	1.35	0.43	0.0000863	0.021297263
RGMA	1840.04	2478.46	1.35	0.43	0.000125448	0.025533716
LAMB2	17914.23	23963.84	1.34	0.42	0.0000334	0.010763391
MBD5	1431.09	1918.57	1.34	0.42	0.00030649	0.043418537
DUSP3	3569.15	4779.36	1.34	0.42	0.000923989	0.083907686
PROS1	4485.99	6012.43	1.34	0.42	0.001136067	0.09534351
LRP10	5794.76	7699.77	1.33	0.41	0.000104939	0.022703107
KCNE4	4544.07	6036.7	1.33	0.41	0.0000982	0.022703107
KALRN	2675.52	3564.68	1.33	0.41	0.000132753	0.025950843
DDR1	2626.31	3500.26	1.33	0.41	0.000137989	0.026559959
ITPKC	1298.2	1720.46	1.33	0.41	0.000490814	0.057558562
RDH10	1024.45	1359.87	1.33	0.41	0.000755847	0.074226372
DKK3	11885.84	15635.32	1.32	0.4	0.00010248	0.022703107
DNMBP	5532.82	7300.37	1.32	0.4	0.000122043	0.02529017
DPF3	1941.25	2556.63	1.32	0.4	0.000394203	0.050952915
TNFRSF21	11641.51	15341.32	1.32	0.4	0.000548971	0.062717271
GBE1	1011	1329.46	1.31	0.4	0.001169815	0.097265226
CREBRF	832.62	1102.36	1.32	0.4	0.001197905	0.098206004
RPS6KA2	12393.4	16280.65	1.31	0.39	0.000108736	0.02319886
RHOB	8341.78	10966.25	1.31	0.39	0.000273639	0.041415378
LYST	2644.46	3473.7	1.31	0.39	0.000286455	0.042342253
PLEKHG1	2289.41	2995.1	1.31	0.39	0.000408861	0.051348327
NPTXR	6891.62	8944.57	1.3	0.38	0.000266677	0.041361015
FRMD5	3102.85	4027.1	1.3	0.38	0.000461542	0.054507908
KIAA0513	1949.34	2538.14	1.3	0.38	0.000617488	0.066673589
VIM-AS1	98961.6	129000.43	1.3	0.38	0.000630753	0.067693149
BAMBI	2020.97	2633.22	1.3	0.38	0.000937934	0.084150337
ENC1	11652.26	15041.36	1.29	0.37	0.00029996	0.043184528
PHLDA1	30286.37	39210.44	1.29	0.37	0.000741707	0.073821636
NMNAT2	1972.33	2546.45	1.29	0.37	0.000933068	0.084150337
EHD2	7049.05	9027.35	1.28	0.36	0.000547828	0.062717271
SFXN3	3940.27	5048.96	1.28	0.36	0.000736923	0.073821636
GLIS3	3059.03	3932.08	1.29	0.36	0.000758695	0.074226372
PFKFB3	3151.34	4043.93	1.28	0.36	0.000827604	0.078370158
HEXA	2643.99	3382.19	1.28	0.36	0.001103886	0.094873677
TPP1	7434.01	9485.46	1.28	0.35	0.000646177	0.068930734
SHC1	4325.65	5525.43	1.28	0.35	0.000791225	0.076562888
CX3CL1	4468.04	5691.91	1.27	0.35	0.000882155	0.081786385
IDS	13878.13	17536.63	1.26	0.34	0.000880096	0.081786385

VIM	199429.16	251432.83	1.26	0.33	0.000812058	0.077729359
CD63	13632.14	17127.4	1.26	0.33	0.001196946	0.098206004
SRPK1	7111.14	5616.32	0.79	-0.34	0.001100202	0.094873677
FDFT1	18744.36	14714.47	0.79	-0.35	0.000586954	0.064557683
ELOVL6	8349.82	6491.66	0.78	-0.36	0.000461686	0.054507908
IDH1	19296.62	14933.56	0.77	-0.37	0.000270944	0.041361015
CCNF	5121.55	3954.64	0.77	-0.37	0.000617382	0.066673589
HMGCS1	51072.73	39166.46	0.77	-0.38	0.000972191	0.086343952
NUDCD1	2983.9	2274.56	0.76	-0.39	0.00038796	0.050514637
ZNF248	2097.71	1606.24	0.77	-0.39	0.000742792	0.073821636
NOC3L	3094.22	2347.36	0.76	-0.4	0.000286936	0.042342253
SKP2	4924.55	3568.77	0.72	-0.46	0.0000449	0.013198583
FANCL	1224.01	877.75	0.72	-0.48	0.000122823	0.02529017
FIGNL1	1220.49	870.78	0.71	-0.49	0.000100709	0.022703107
RMI2	1244.61	871.69	0.7	-0.51	0.0000451	0.013198583
AMOT	10207.06	7007.39	0.69	-0.54	0.001098561	0.094873677
FANCM	1590.5	1049.95	0.66	-0.6	0.000026	0.009291451
FAAP24	267.1	167.66	0.63	-0.67	0.000568932	0.063362616
SEC11C	1191.11	694.41	0.58	-0.78	0.0000473	0.013282286

Table S5. Common genes that are differentially regulated in brain tumor-initiating cells following FOXG1 or TLE1 knockdown and have proximal FOXG1 binding sites.
Genes that are differentially expressed following FOXG1 or TLE1 knockdown and have proximal FOXG1 binding sites are listed based on RNA-seq data.

id	baseMean.Scr	baseMean.FOXG1	foldChange.FOXG1	log2FoldChange.FOXG1	pval.FOXG1	padj.FOXG1	baseMean.Scr	baseMean.TLE	foldChange.TLE	log2FoldChange.T	pval.TLE	padj.TLE
MIR30A	2598	8669	3.34	1.74	0.000513	0.059739	2598	10223	3.94	1.98	0.000089	0.007813
BMF	14	41	3.03	1.6	0.000746	0.073822	14	47	3.49	1.8	0.000115	0.009364
MCHR1	392	1182	3.02	1.59	0	0	392	919	2.34	1.23	0	0.000006
STX3	27	81	3.01	1.59	0.000005	0.002986	27	73	2.7	1.44	0.000047	0.00516
NEK10	38	108	2.85	1.51	0.000046	0.013199	38	110	2.89	1.53	0.000036	0.00423
SLC7A11	327	925	2.83	1.5	0.000617	0.066674	327	1434	4.38	2.13	0.000002	0.000452
AHNAK2	54	148	2.76	1.46	0	0.000051	54	136	2.53	1.34	0	0.00015
SERPINE1	1068	2840	2.66	1.41	0.000057	0.015338	1068	2497	2.34	1.23	0.000452	0.02244
GDF15	716	1836	2.56	1.36	0	0.000243	716	1690	2.36	1.24	0.000002	0.00042
GPNMB	54	128	2.39	1.25	0.000006	0.002986	54	107	2	1	0.00037	0.0195
ABCA12	111	257	2.31	1.21	0.000011	0.004916	111	319	2.87	1.52	0	0.000019
CDH13	66	147	2.24	1.16	0.000011	0.005132	66	122	1.86	0.89	0.000867	0.033507
HIST1H2BD	42	92	2.2	1.14	0.000241	0.040429	42	93	2.22	1.15	0.000191	0.013376
PLA2G4C	93	204	2.19	1.13	0.000004	0.002549	93	249	2.68	1.42	0	0.000005
CHRNA9	156	335	2.15	1.11	0	0.000015	156	353	2.27	1.18	0	0.000001
BLVRB	69	141	2.06	1.04	0.000068	0.017507	69	123	1.8	0.85	0.001378	0.044131
RBM47	181	369	2.04	1.03	0.000044	0.013199	181	378	2.09	1.07	0.000024	0.003207
NR4A3	161	326	2.02	1.02	0.00013	0.025951	161	302	1.87	0.91	0.000648	0.028256
HAP1	733	1363	1.86	0.9	0	0	733	1706	2.33	1.22	0	0
MARCH3	78	144	1.85	0.89	0.000387	0.050515	78	132	1.69	0.76	0.0025	0.062521
KCNB1	142	250	1.77	0.82	0.000026	0.009291	142	282	1.99	0.99	0	0.000122
RGS16	1664	2938	1.77	0.82	0.000118	0.024924	1664	2684	1.61	0.69	0.001183	0.040513
BEST1	104	183	1.76	0.81	0.000246	0.040429	104	195	1.87	0.9	0.000041	0.004606
MAP6	254	446	1.76	0.81	0	0.000247	254	721	2.84	1.51	0	0
CNIH3	853	1491	1.75	0.8	0.000013	0.005302	853	1438	1.69	0.75	0.000044	0.004919
ELL2	96	166	1.73	0.79	0.000673	0.070147	96	155	1.61	0.69	0.003364	0.074326
LONRF2	1884	3258	1.73	0.79	0.000824	0.07837	1884	4430	2.35	1.23	0	0.000093
RND3	1008	1690	1.68	0.75	0	0.000034	1008	1320	1.31	0.39	0.003105	0.070754
AKAP6	653	1093	1.67	0.74	0.000002	0.001429	653	1824	2.79	1.48	0	0
FOSL2	1187	1956	1.65	0.72	0.000003	0.001854	1187	1843	1.55	0.63	0.000035	0.004115
KLF5	215	354	1.65	0.72	0.000431	0.052982	215	354	1.65	0.72	0.000433	0.021741
KANK3	179	294	1.64	0.72	0.000148	0.027463	179	313	1.74	0.8	0.00002	0.002916
METTL7B	2121	3464	1.63	0.71	0	0.00004	2121	3105	1.46	0.55	0.000012	0.002065
NUAK2	286	467	1.63	0.71	0.000009	0.004424	286	429	1.5	0.58	0.000257	0.015616
GPR37	171	273	1.6	0.68	0.000363	0.048638	171	249	1.45	0.54	0.004619	0.089138
SLC35D2	721	1144	1.59	0.67	0.000661	0.069431	721	1056	1.46	0.55	0.004949	0.093245
ABTB2	1117	1714	1.54	0.62	0.000299	0.043185	1117	2295	2.06	1.04	0	0.000002
NRP2	15538	23852	1.54	0.62	0.000002	0.001711	15538	28186	1.81	0.86	0	0
TMEM59L	544	837	1.54	0.62	0.000735	0.073822	544	806	1.48	0.57	0.002141	0.057355
NEDD9	2681	4097	1.53	0.61	0.000941	0.08415	2681	4053	1.51	0.6	0.001274	0.042021
HID1	323	492	1.52	0.61	0.00008	0.020163	323	504	1.56	0.64	0.00003	0.003788
RIN1	658	1000	1.52	0.6	0.000009	0.004424	658	1132	1.72	0.78	0	0.000007
HIVEP3	1825	2746	1.5	0.59	0.00015	0.027463	1825	2972	1.63	0.7	0.000006	0.00119
FAM84B	3004	4495	1.5	0.58	0.000042	0.013199	3004	4965	1.65	0.72	0	0.000135
SLC22A4	294	440	1.5	0.58	0.000256	0.040708	294	425	1.45	0.53	0.000872	0.033536
ORAI3	443	655	1.48	0.57	0.00015	0.027463	443	625	1.41	0.5	0.000899	0.034064
SERPINE2	4960	7365	1.48	0.57	0	0.000087	4960	6423	1.3	0.37	0.000359	0.019233
FGF1	366	535	1.46	0.55	0.000198	0.034447	366	703	1.92	0.94	0	0
OSBP2	2633	3837	1.46	0.54	0.000001	0.000618	2633	3318	1.26	0.33	0.002127	0.057161
EFNA1	517	750	1.45	0.54	0.000104	0.022703	517	684	1.32	0.4	0.003702	0.077903
RNF19A	11010	15705	1.43	0.51	0.000001	0.00059	11010	16462	1.5	0.58	0	0.00001
GRIK2	667	947	1.42	0.51	0.000103	0.022703	667	926	1.39	0.47	0.000282	0.016775
COL4A1	42223	59636	1.41	0.5	0.000001	0.000727	42223	61368	1.45	0.54	0	0.000041
PROX1	4567	6422	1.41	0.49	0.00001	0.004916	4567	6728	1.47	0.56	0.000001	0.000195
ALK	4252	5959	1.4	0.49	0.000663	0.069431	4252	8172	1.92	0.94	0	0
CRIM1	3346	4701	1.4	0.49	0.000004	0.002802	3346	4494	1.34	0.43	0.000067	0.006587
MAPK10	1042	1462	1.4	0.49	0.000046	0.013199	1042	1637	1.57	0.65	0	0.000029
MATN2	9004	12624	1.4	0.49	0.000002	0.001584	9004	12449	1.38	0.47	0.000005	0.001024
SNPH	941	1310	1.39	0.48	0.000097	0.022703	941	1396	1.48	0.57	0.000003	0.000707
SYTL5	454	633	1.39	0.48	0.000808	0.077729	454	633	1.39	0.48	0.000799	0.031779
NOS1AP	659	915	1.39	0.47	0.000296	0.043185	659	980	1.49	0.57	0.000012	0.001997
PLCD3	2796	3872	1.38	0.47	0.000015	0.005875	2796	4061	1.45	0.54	0.000001	0.000223
MARCH2	490	675	1.38	0.46	0.000975	0.086344	490	689	1.41	0.49	0.000447	0.022226
SLC9A7	696	960	1.38	0.46	0.000332	0.046109	696	990	1.42	0.51	0.000083	0.00763
AMIGO2	596	819	1.37	0.46	0.000555	0.062986	596	975	1.63	0.71	0	0.000041
C1orf198	2596	3563	1.37	0.46	0.000027	0.009291	2596	3301	1.27	0.35	0.001434	0.045446
C1orf226	1068	1462	1.37	0.45	0.000153	0.027585	1068	1615	1.51	0.6	0.000001	0.000204
GPC1	25273	34308	1.36	0.44	0.000012	0.005168	25273	32471	1.28	0.36	0.000331	0.018263
MAN2B2	2372	3215	1.36	0.44	0.000062	0.016368	2372	3232	1.36	0.45	0.000046	0.005125
P4HTM	1031	1399	1.36	0.44	0.000269	0.041361	1031	1450	1.41	0.49	0.000047	0.00516
RGMA	1840	2478	1.35	0.43	0.000125	0.025534	1840	2539	1.38	0.46	0.000033	0.003966
SHROOM2	2498	3375	1.35	0.43	0.000086	0.021297	2498	3205	1.28	0.36	0.001139	0.039325
DUSP3	3569	4779	1.34	0.42	0.000924	0.083908	3569	4740	1.33	0.41	0.001285	0.042287
LAMB2	17914	23964	1.34	0.42	0.000033	0.010763	17914	24465	1.37	0.45	0.000009	0.001573
MBD5	1431	1919	1.34	0.42	0.000306	0.043419	1431	1869	1.31	0.38	0.00102	0.036567
PROS1	4486	6012	1.34	0.42	0.001136	0.095344	4486	5951	1.33	0.41	0.001693	0.049886
DDR1	2626	3500	1.33	0.41	0.000138	0.02656	2626	3403	1.3	0.37	0.000586	0.026672
ITPKC	1298	1720	1.33	0.41	0.000491	0.057559	1298	1954	1.51	0.59	0	0.000152
KALRN	2676	3565	1.33	0.41	0.000133	0.025951	2676	3889	1.45	0.54	0.000001	0.000204
LRP10	5795	7700	1.33	0.41	0.000105	0.022703	5795	7957	1.37	0.46	0.000015	0.00241
RDH10	1024	1360	1.33	0.41	0.000756	0.074226	1024	1360	1.33	0.41	0.000745	0.030471
CREBRF	833	1102	1.32	0.4	0.001198	0.098206	833	1250	1.5	0.59	0.000003	0.000601
DKK3	11886	15635	1.32	0.4	0.000102	0.022703	11886	14582	1.23	0.29	0.003793	0.078659
DNMBP	5533	7300	1.32	0.4	0.000122	0.02529	5533	7234	1.31	0.39	0.000203	0.013655
GBE1	1011	1329	1.31	0.4	0.00117	0.097265	1011	1320	1.31	0.38	0.001569	0.047807
LYST	2644	3474	1.31	0.39	0.000286	0.042342	2644	3768	1.42	0.51	0.000003	0.000593
PLEKHG1	2289	2995	1.31	0.39	0.000409	0.051348	2289	3480	1.52	0.6	0	0.000022
RPS6KA2	12393	16281	1.31	0.39	0.000109	0.023199	12393	16758	1.35	0.44	0.000019	0.002798
FRMD5	3103	4027	1.3	0.38	0.000462	0.054508	3103	3894	1.25	0.33	0.002312	0.059599
KIAA0513	1949	2538	1.3	0.38	0.000617	0.066674	1949	3029	1.55	0.64	0	0.000009
NPTXR	6892	8945	1.3	0.38	0.000267	0.041361	6892	9516	1.38	0.47	0.000007	0.001231
ENC1	11652	15041	1.29	0.37	0.0003	0.043185	11652	16919	1.45	0.54	0	0.000061
NMNAT2	1972	2546	1.29	0.37	0.000933	0.08415	1972	2862	1.45	0.54	0.000001	0.000388
GLIS3	3059	3932	1.29	0.36	0.000759	0.074226	3059	4013	1.31	0.39	0.00027	0.016097
EHD2	7049	9027	1.28	0.36	0.000548	0.062717	7049	8764	1.24	0.31	0.002349	0.059818
SHC1	4326	5525	1.28	0.35	0.000791	0.076563	4326	5823	1.35	0.43	0.000047	0.00516
TPP1	7434	9485	1.28	0.35	0.000646	0.068931	7434	10002	1.35	0.43	0.000033	0.003953
CX3CL1	4468	5692	1.27	0.35	0.000882	0.081786	4468	6423	1.44	0.52	0.000001	0.000204
IDS	13878	17537	1.26	0.34	0.00088	0.081786	13878	19666	1.42	0.5	0.000001	0.000231
FDFT1	18744	14714	0.79	-0.35	0.000587	0.064558	18744	14122	0.75	-0.41	0.000059	0.006046
CCNF	5122	3955	0.77	-0.37	0.000617	0.066674	5122	3949	0.77	-0.38	0.000579	0.02643
IDH1	19297	14934	0.77	-0.37	0.000271	0.041361	19297	14714	0.76	-0.39	0.000117	0.009366
HMGCS1	51073	39166	0.77	-0.38	0.000972	0.086344	51073	37929	0.74	-0.43	0.000221	0.014555
SKP2	4925	3569	0.72	-0.46	0.000045	0.013199	4925	3186	0.65	-0.63	0	0.000022
FIGNL1	1220	871	0.71	-0.49	0.000101	0.022703	1220	930	0.76	-0.39	0.001689	0.049886
RMI2	1245	872	0.7	-0.51	0.000045	0.013199	1245	767	0.62	-0.7	0	0.000022
